# Associations of potentially inappropriate medications in older adults with mortality and hospitalizations: methodological challenges in pharmacoepidemiology

**DOI:** 10.1007/s10654-025-01294-x

**Published:** 2025-08-23

**Authors:** Miriam Degen, Ben Schöttker

**Affiliations:** 1 Division of Clinical Epidemiology and Aging Research, German Cancer Research Center (DKFZ), Im Neuenheimer Feld 581, 69120 Heidelberg, Germany; 2 Faculty of Medicine, University of Heidelberg, 69115 Heidelberg, Germany

**Keywords:** Pharmacoepidemiology, Drug safety, Potentially inappropriate medication, Clinicalpharmacology

## Abstract

Previous studies on the association of potentially inappropriate medication (PIM) use with hospitalization risk and all-cause mortality among older adults were prone to confounding and biases. Using data from 217,111 participants of the population-based United Kingdom Biobank, aged 60–69 years, including 95,187 participants with primary care data linkage, the main analysis was a prospective new user design with 1:1 propensity-score stratified by indication matching of new PIM users and new appropriate medication (AM) users (assessed with the EURO-FORTA list). Results were compared to previous approaches with a prevalent user design and a new user design without propensity score matching. 43,307 (19.9%) participants used at least one PIM at baseline. Among 11,812 propensity score matched individuals with new PIM or new AM prescription within 2 years after baseline, new PIM use was associated with non-significantly 20% increased 1-month hospitalization (hazard ratio (HR) [95% confidence interval (95% CI)]: 1.20 [0.76–1.90]) and 23% increased 1-year mortality (1.23 [0.80–1.89]). Null-results were obtained with the prevalent user design (HRs [95% CIs]: 1-month hospitalization: 1.04 [0.83–1.31]; 1-year mortality: 1.01 [0.82–1.23]) and slightly stronger associations in new user design without propensity score matching stratified by indication (1-month hospitalization (1.24 [0.95–1.61]); 1-year mortality (HR [95% CI] 1.57 [1.24–2.00]). This first study with an appropriate methodology showed that previous pharmacoepidemiologic studies on the risk of PIM for hospitalization and mortality have either under- or overestimated the risk. Effect sizes of about 20% appear biologically plausible and larger studies are needed to detect such weak associations with statistical significance.

**Supplementary Information:**

The online version contains supplementary material available at 10.1007/s10654-025-01294-x.

## Introduction

Ageing is a global challenge, with the proportion of people aged over 60 expected to double by 2050 [1]. Consequently, the prevalence of diseases has risen considerably, largely due to advancements in treatments that prolong the lives of affected individuals [2].

Certain drugs, known as potentially inappropriate medications (PIM), are considered unsuitable for older patients due to a poor benefit-harm balance and available safer alternatives. The risks associated with PIM use might vary by setting and population, with pronounced effects in vulnerable populations such as frail, very old or hospitalised patients. A variety of PIM assessment tools have been developed [3–10], primarily through consensus-driven processes. These tools differ in structure and content; for instance, negative lists, such as Beers criteria [3], specifically identify medications that should be avoided. In contrast, tools like EURO-FORTA [4] not only recommend alternative medications but also address overuse and underuse of medications, which collectively contribute to suboptimal prescribing.

While randomized controlled trials (RCTs) would ideally compare new prescriptions of PIM to the new prescription of alternative medications, such trials are difficult to conduct not only due to ethical considerations. The available RCTs aimed to reduce prevalent PIM use, which has not shown clear improvements in clinical outcomes [11]. The likely explanation is that the included study participants with PIM tolerate them well. Otherwise, they would have switched to other drugs before. Previous observational studies, aiming to provide evidence for hospitalization and mortality rate reductions by avoiding PIM in older adults, yielded inconsistent results [12–14].

Observational studies are essential for deriving evidence on drug safety from real-world settings. Nonetheless, specific methodological challenges in pharmacoepidemiology, such as immortal time bias, healthy-adherer bias, depletion of susceptibles, and confounding by indication, have been observed to affect the judgement of the risk and benefit profiles associated with certain medications in the past [15–21]. Since such findings may influence treatment strategies, policymaking, and subsequent research efforts, minimizing such biases is crucial. The meta-analysis by Muhlack et al., which addressed time-dependent biases, revealed a statistically significant 1.6-fold increased risk for all-cause mortality among PIM users in *n* = 3 observational studies with a new user design. However, in their meta-analysis of *n* = 10 studies with prevalent user design, no increased mortality among PIM users was detected [14].

Given the methodological challenges of pharmacoepidemiologic studies and the large implications for medication use among vulnerable older patients, we aim to (1) assess the association of PIM with the risk of hospitalizations and all-cause mortality in community-dwelling older adults avoiding the aforementioned sources of bias, and to (2) elucidate how common previous study designs might have under- or overestimated the risks associated with PIM in previous studies.

## Materials and methods

This study was reported according to RECORD-PE [22].

### Data source

We used data from the UK Biobank, a comprehensive prospective cohort that recruited over 500,000 participants aged 40 to 69 years from England, Scotland, and Wales between 2006 and 2010 [23]. During the baseline visit at one of the 22 assessment centres, participants gave electronically signed consent, and participated a brief interview, touch-screen questionnaires, and multiple physical and functional assessments [23, 24]. Information on prescription medication information was collected by nurse-led verbal interviews. Participants were asked to bring their medications to the assessment centres and those taken regularly (weekly, monthly, or three-monthly use) were recorded [25, 26]. Over-the-counter medications, vitamins, and supplements were documented in the touch-screen questionnaire [27].

Linked electronic health records included the UK National Health Service (NHS) data, primary care records including general practitioner prescriptions (available for approximately half of the cohort), hospital inpatient data, and death registries [28].

The study adhered to the principles outlined in the Declaration of Helsinki. The UK Biobank was approved by the North West Multi-center Research Ethics Committee (approval ID 2016: #16/NW/027; renewed in 2021: 21/NW/0157).

### Study population

**Fig. 1:**
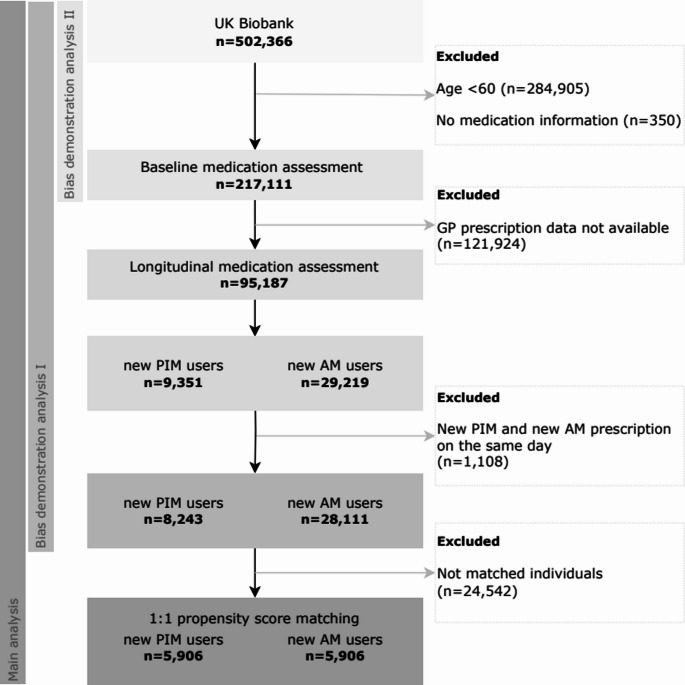
Flow chart of the study population. *AM* appropriate medication, *GP* general practitioner, *PIM* potentially inappropriate medication

From 502,366 UK Biobank participants, we excluded *n* = 284,905 who were younger than 60 years and *n* = 350 without available medication information at baseline assessment, leaving *n* = 217,111 individuals (Fig. 1), of whom the majority (98.9%) was aged 60–69 years, and only a small minority was up to 73 years old. In the main analysis, we additionally excluded participants without primary care linkage, leaving *n* = 95,187 individuals with longitudinal medication assessment.

### Definition of potentially (in-)appropriate medication

PIM and AM were defined in line with the EURO-FORTA (Fit fOR The Aged) List Version 2 [4], that considers different medication availability and prescribing practices in European countries and is regularly updated. The safety, efficacy, and appropriateness of medications for frequent geriatric indications are categorized into four categories, from A (*indispensable*) to D (*avoid*). This assessment tool was chosen because it does not only list PIM but also lists appropriate medication (AM) for each indication that aligned with consensus recommendations and served as the active comparator in our study. EURO-FORTA classes A-B were defined as AM and classes C-D as PIM, respectively. Read v2 codes, used for coding the prescription data by the UK Biobank, were translated into ATC codes. A list of the ATC codes used to code PIM and AM according to the EURO-FORTA list is provided in Table S1 (Suppl. Material). Short-term use of glucocorticoids in patients with acute exacerbation of chronic obstructive lung disease could not be coded as AM and long-term use of NSAIDs could not be coded as PIM because of missing data for the duration of drug use in the UK Biobank. Thus, these two drug classes were not considered in the main PIM/AM assessment, but we explored the impact of excluding NSAIDs on the results in a sensitivity analysis.

Cancer indications of the FORTA list were not used in this population-based cohort. Furthermore, we needed to restrict the main analysis to those 14 FORTA indications, for which both study participants who use PIM and who use AM could be identified in the UK Biobank. For some medications, it was possible to assign them to more than one FORTA indication. To limit the assignment of each drug to only one FORTA indication, a few rules needed to be specified (Methods S1 (Suppl. Material)).

### Outcome ascertainment

Hospitalization dates were derived from linkage to hospital inpatient data (data fields ‘41280’ and ‘41281’, referring to the *first date of in-patient diagnosis (ICD10 or ICD-9)*). Information about vital status and date of death was assessed through linkage with national death registries. Both linkages were available for all participants. Follow-up data were completely available for mortality until 19/12/2022 and hospitalizations until 31/05/2022. This covers more than the needed up to 4-year follow-up period from the last recruited UKB participant (01/10/2010) plus the 2-year window after baseline recruitment for prescription of a new PIM or AM (i.e., the last theoretical cohort entry for the new user design analysis was 01/10/2012). Participants alive at the end of each follow-up period used in this investigation were censored.

### Covariates ascertainment

To account for potential confounders [21, 29], we incorporated *n* = 41 covariates (Table S2 (Suppl. Material)), encompassing sociodemographic and economic factors, lifestyle factors, diseases (e.g., diabetes, cancer), functional assessments, and blood-based biomarkers (e.g., low-density lipoprotein (*LDL*), HbA_1c_).

Sociodemographic (e.g., average household income) and lifestyle (e.g., smoking status, alcohol consumption, and physical activities) information was gathered through touchscreen questionnaires [27]. Self-reported medical history was obtained through touchscreen questionnaires and verbal interviews [27, 30], and completed by diagnoses obtained through linkage to primary care records and hospital inpatient data. Physical examinations (e.g., blood pressure measurements) and laboratory methods for blood-based biomarkers are detailed in another publication [31].

### Statistical analyses

#### General remarks

We used SAS statistical software (version 9.4, SAS Institute, Inc., Cary, NC, USA) to conduct the statistical analyses, and tested the assumption of proportional hazards through Schoenfeld residuals. Only previous smoking violated the assumption for mortality outcomes, thus its interaction term with follow-up time was included in the model. To assess hospitalization, cause-specific Cox regression models were used to account for the competing risk of death. All statistical tests were two-tailed, with a significance level set at α = 0.05. We imputed missing data using multiple imputation with the fully conditional specification method [32]. Only three variables had missing value proportions > 4.1% (physical activity (25.9%), income (19.4%), and years since quitting smoking (13.3%). We explored potential sources of non-random missingness, revealing a higher proportion of women and lower proportion of participants with higher incomes among participants with missing values for physical activity compared to the total study population. As sex and income were incorporated in the imputation model, missing values can be assumed to be missing at random. We generated 10 imputed datasets and aggregated analytical outcomes from the imputed datasets using the SAS procedure ‘PROC MIANALYZE’. Proportions of missing values prior to imputation are detailed in Table S2 (Suppl. Material).

#### The association of PIM with the study outcomes

Our primary outcomes were 1-month hospitalization and 1-year all-mortality as these were the shortest follow-up times at which ≥ 100 events could be included in the main analysis. In sensitivity analyses, we explored longer observation periods (up to 4 years). We used this approach to address two competing needs: our study outcomes were acute events that predominantly occur during the early treatment period which required short-term follow-ups. However, this could reduce the statistical power due to fewer observed events.

**Fig. 2:**
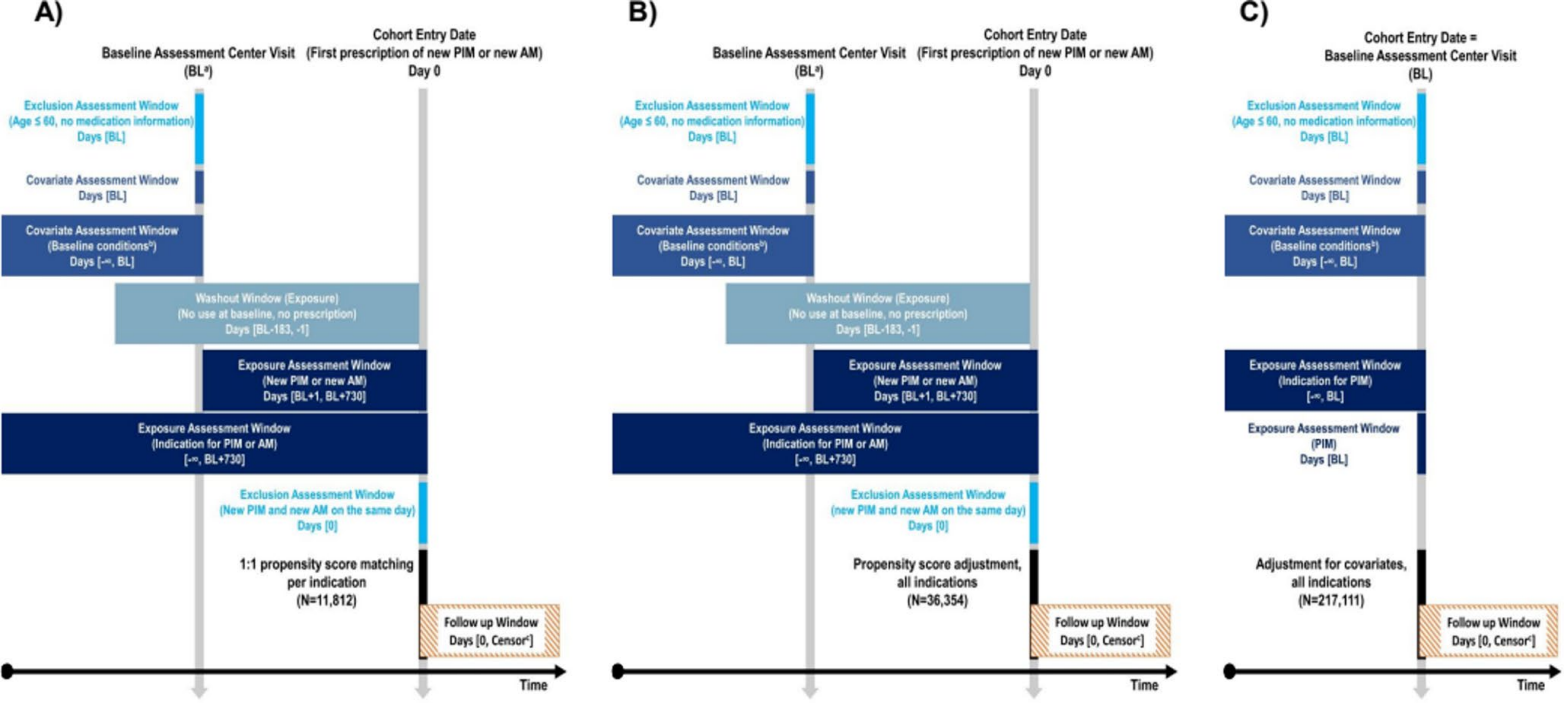
Visualization of the three study designs. Main analysis: active comparator propensity score matching per indication (**A**), bias demonstration analysis I: new user design with propensity score adjustment instead of matching by indication (**B**), bias demonstration analysis II: prevalent user design. *AM* appropriate medication, *BL* baseline assessment centre visit, *PIM* potentially inappropriate medication. ^a^Maximum time between baseline assessment centre visit and cohort entry: 730 days. ^b^Diagnoses from primary care and hospital records were added to achieve complete information. ^c^At earliest of outcome or end of the follow-up period

Three distinct study designs were applied and visualized in Fig. 2 [33]: The main analysis with a new user design and active comparator matching per indication (Fig. 2A) compared new PIM and new AM users identified through general practitioner prescription data. We assessed prescriptions from day 1 after the baseline visit at the study assessment centre until 2 years after baseline, with the first date of a new PIM/AM prescription being the index date. We restricted the exposure assessment window to 2 years after baseline because the accuracy of the covariates could not be assumed over a longer period. We verified new PIM/AM by implementing a 6-month washout window. This means that medications taken at baseline, as reported during the baseline assessment center visit, or prescribed in the six months leading up to the baseline assessment visit were not considered new medications. To determine possible PIM/AM indications, we used self-reported information gathered at the baseline assessment centre visit and completed them by medical diagnoses from primary care and hospital inpatient records. Since these were mainly chronic conditions, we reviewed all available history of medical diagnoses prior to the baseline visit and up to the end of the 2-year exposure assessment window. Participants who were simultaneously prescribed with new PIM and new AM were excluded.

We assigned new PIM and new AM users to the indication-specific cohort depending on the relevant indication. We calculated distinct propensity scores per indication for receiving new PIM versus new AM, with detailed methods outlined in Methods S2 (Suppl. Material). We subsequently matched new PIM and new AM users of the same indication in a 1:1 ratio using the SAS procedure ‘PROC PSMATCH’. Matched pairs were then pooled into one analytical dataset that included all indications. New PIM and new AM users had well-balanced covariates in this matched dataset (Table S3 (Suppl. Material)). We analysed the pooled matched dataset using Cox proportional hazards regression without any adjustment because confounding is controlled for by the propensity score matching.

*Bias demonstration analysis I* applied a new user design without appropriate active comparator selection (Fig. 2B). New PIM and new AM users were identified using the same identification process as applied in the main analysis. In contrast to the main analysis, we did not distinguish between the different PIM/AM indications. All identified new PIM/AM users were included into the same analytical dataset, which was much larger (*N* = 36,354) than in the main analysis with active comparator design (*N* = 11,812).

The association of new PIM versus new AM use with the study outcomes was assessed using two distinct multivariable Cox proportional hazard regression models: model 1, which was adjusted for age and sex, and model 2, which was adjusted for a propensity score. Only one propensity score was calculated for the whole study population with the ß coefficients obtained from a logistic regression model with receiving new PIM versus new AM of any indication as the dependent and all *n* = 41 covariates as independent variables. The propensity score was fairly distributed between new PIM and new AM users (Figures S1–S5 (Suppl. Material)), and all covariates were well-balanced after propensity score adjustment (Table S4 (Suppl. Material)).

*Bias demonstration analysis II* employed a prevalent PIM user versus non-user approach (Fig. 2C). Therefore, we identified PIM users (≥ 1 PIM) and non-users (no PIM) through an assessment on their baseline medication information. The baseline medication information included the medication information gathered at the baseline visit. We used self-reported information gathered at the baseline assessment centre visit and diagnoses identified through primary care and hospital inpatient records from the full available period prior to baseline to identify possible PIM indications.

The association of PIM use versus non-use with the study outcomes was assessed using two distinct multivariable Cox proportional hazard regression models: model 1, which was adjusted for age and sex, and model 2, which was adjusted for all *n* = 41 covariates. As no matching was involved, the total cohort was included (*N* = 217,111).

All analyses applied an intention-to-treat principle assuming adherence to the assigned treatment group, because the data did not allow to measure adherence, which also aligns with the nature of prescribing in primary care.

#### Sensitivity analysis including NSAIDs

The EURO-FORTA list only defines chronic intake of NSAIDs as PIM but the duration of the use of each drug was not recorded in the UKB dataset [4]. As this information was not available, we decided not to include NSAIDs use in the main analysis and conducted a sensitivity analysis including NSAIDs instead. As the baseline interview information about NSAIDs use was needed to determine the chronicity of NSAIDs use (as NSAIDs are usually used as OTC drugs, and often are not recorded in prescription data), this sensitivity analysis could only be conducted for the prevalent PIM user analysis (*Bias demonstration analysis II*). In this sensitivity analysis, we assumed that NSAIDs were used regularly, and only coded them as PIM, if NSAID use was reported in the baseline interview and additionally at least one of the following 3 conditions was met:

1. Participants answered “Yes” to the interview question about regular ibuprofen or aspirin use.
2. Participants had at least one NSAID prescription within 60 days prior baseline according to the primary care prescription data.
3. Participants had at least two NSAID prescriptions within 120 days before baseline according to the primary care prescription data.

#### Subgroup analyses

We conducted subgroup analyses by age (< 65/≥ 65 years of age), sex (female/male), polypharmacy (yes, defined by the concurrent intake of ≥ 5 medications/no), and prevalent PIM use at baseline (yes/no). We repeated the matching process of the main analysis in each subgroup. We used the shortest follow-up period at which it was possible to include ≥ 100 events in all subgroups. However, as the maximum follow-up time should not be more than 1 year, this number of events were not available for the mortality outcome. Thus, the subgroup analyses were only conducted for the outcome 6-months all-cause hospitalizations.

## Results

### Characteristics of the study population

The baseline characteristics of the study population (*n* = 217,111) are presented in Table 1. The median age was 64 (interquartile range (IQR): 62–66) years at baseline, slightly more participants were female (52.8%) and 43,307 (19.9%) participants used at least one PIM at baseline. Pain (70.4%), arterial hypertension (38.3%), and gastrointestinal illnesses (28.8%) were the most frequent conditions.

Out of 95,187 participants with primary care data linkage, 9,351 (9.8%) participants received newly prescribed PIM and 29,219 (30.7%) got new AM prescriptions within 2 years after baseline. After exclusion of 1108 participants receiving both prescription types on the same day, 8243 new PIM and 28,111 new AM users were eligible for the propensity score matching. The main analysis included 11,812 individuals, consisting of 5,906 new PIM and 5,906 new AM users matched in a 1:1 ratio (Fig. 1). The comparison of the patient characteristics of these two groups is presented in Table S5 (Suppl. Material) and all were well balanced.

**Table 1:** Baseline characteristics of the population analyzed (*N* = 217,111)

Characteristics	*N* (%)^a^	Median (IQR)^a^
Sociodemographic/-economic factors		
Sex	–	–
Female	114,535 (52.8)	
Male	102,576 (47.2)	
Age		64 (62; 66)
Income^b^	–	–
Very low	77,606 (35.7)	
Low	70,875 (32.6)	
Middle	43,366 (20.0)	
High	20,417 (9.4)	
Very high	4,874 (2.2)	
Years of schooling	–	
≤ 10	114,282 (52.6)	
11–<14	32,055 (14.8)	
≥ 14	70,774 (32.6)	
Lifestyle factors		
BMI (kg/m^2^)		27.0 (24.5; 30.0)
Physical activity (MET-min/week)		2,085 (1036; 4230)
Alcohol consumption (g/day)		15.0 (0.0; 31.6)
Smoking status	–	–
Never smoker	108,966 (50.1)	
Former smoker, years since quit		
< 5 years	72,868 (33.6)	
5–10 years	1,689 (0.8)	
11–15 years	1,387 (0.6)	
16–20 years	1,716 (0.8)	
> 20 years	12,597 (5.8)	
Current smoker^c^		
Regular	13,385 (6.2)	
Occasional	4,503 (2.1)	
Comorbidities	–	–
Diabetes		
No	201,703 (92.9)	
Untreated	4,345 (2.0)	
Treated with oral antidiabetics^d^	8,102 (3.7)	
Treated with insulin	2,961 (1.4)	
Arterial hypertension	83,050 (38.3)	
Stroke	6,090 (2.8)	
Myocardial infarction	14,591 (6.7)	
Heart failure	2,167 (1.0)	
Acute coronary syndromes	261,77 (12.1)	
Atrial fibrillation	5,573 (2.6)	
Asthma	27,876 (12.8)	
COPD	13,090 (6.0)	
Arthritis	38,406 (17.7)	
Osteoporosis	10,323 (4.8)	
History of fractures^e^	21,340 (9.8)	
Pain^f^	152,811 (70.4)	
Parkinson’s disease	828 (0.4)	
Dementia	2,051 (0.9)	
Depression	25,383 (11.7)	
Bipolar disorder	802 (0.4)	
Insomnia	66,866 (30.8)	
Epilepsy	1,478 (0.7)	
Gastrointestinal illness^g^	62,429 (28.8)	
Incontinence	2,487 (1.1)	
Anaemia	4,153 (1.9)	
History of cancer	28,003 (12.9)	
Overall health rating^h^	–	
Excellent	3,2697 (15.0)	
Good	128,214 (59.1)	
Fair	47,051 (21.7)	
Poor	9,149 (4.2)	
Functional assessments	–	–
Systolic blood pressure (mmHg)		145.9 (19.8)
Diastolic blood pressure (mmHg)		82.4 (10.6)
Grip strength *(kg)*		30.0 (22.0; 39.0)
eGFR^i^ (ml/min/1.73 m^2^)		2 (1; 5)
Blood-based biomarkers	–	–
CRP (mg/L)		1.5 (0.8; 3.0)
HDL (mmol/L)		1.4 (1.2; 1.7)
LDL (mmol/L)		3.5 (2.9; 4.2)
HbA_1c_ (mmol/L)		36.3 (33.9; 39.0)

*BMI* body mass index, *COPD* chronic obstructive pulmonary disease, *CRP* c-reactive protein, *eGFR* estimated glomerular filtration rate, *HbA*_*1c*_ haemoglobin A_1c_, *HDL* high-density lipoprotein, *IQR* interquartile range, *LDL* low-density lipoprotein, *MET* metabolic equivalent of task

^a^Calculated from first imputed dataset

^b^Average total household income before tax. Categories: very low: <18,000£, low: 18,000–30,999£, middle: 31,000–51,999£, high: 52,000–100,000£, very high: >100,000£

^c^Regular smoking was defined as minimum one cigarette, cigar, pipe, etc. per day

^d^Including parenteral formulations of glucagon-like-peptide-1 receptor agonists

^e^Fractures in last five years

^f^Combination of pain-related diagnoses (including migraine, other headache syndromes, facial pain, chronic backpain, chronic pain syndromes, arthritis, osteoarthritis, urinary tract stones, and gout) and self-reported pain in the last month

^g^Including regular use of non-steroidal anti-inflammatory drugs

^h^Self-reported

^i^Calculated based on CKD-EPI equation

### Main analysis: 1:1 new user design with active comparator propensity score matching per indication

Table 2 shows the associations of new PIM use versus new AM use with hospitalization and all-cause mortality. Within one month after cohort entry, 106 participants (0.9%) were hospitalized and 132 patients (1.1%) died during the 1-year follow-up period. The risk for 1-month hospitalization was non-significantly increased by 20% (HR [95% CI] 1.20 [0.76–1.90]). It remained stable with 3 months of follow-up time but decreased afterwards: with follow-up times of 1 year or more, no association was observed. Similarly, we observed a non-significant 23% increased risk for 1-year all-cause mortality (HR [95% CI] 1.23 [0.80–1.89]), but no association between new PIM use and mortality using follow-up times of 2 years or more.

**Table 2:** Main analysis: the associations of new PIM use versus new AM use with hospitalization and all-cause mortality in 1:1 propensity-score matched population (N_total_=11,812)

Outcome	*N*_Event_ (%)^a^	HR (95% CI)^b^	*p*
	PIM vs. AM users		
Hospitalization			
1 month	55 (0.9) vs. 51 (0.9)	1.20 (0.76; 1.90)	0.439
3 months	102 (1.7) vs. 91 (1.5)	1.20 (0.88; 1.62)	0.247
6 months	247 (4.2) vs. 219 (3.7)	1.10 (0.90; 1.34)	0.344
1 year	422 (7.1) vs. 411 (7.0)	1.03 (0.87; 1.22)	0.748
2 years	764 (12.9) vs. 735 (12.4)	1.03 (0.91; 1.16)	0.678
3 years	1,130 (19.1) vs. 1052 (17.8)	1.03 (0.92; 1.16)	0.550
4 years	1,486 (25.2) vs. 1,415 (24.0)	1.02 (0.91; 1.14)	0.722
All-cause mortality^c^			
1 year	69 (1.2) vs. 63 (1.1)	1.23 (0.80; 1.89)	0.347
2 years	106 (1.8) vs. 116 (2.0)	1.00 (0.72; 1.39)	0.998
3 years	153 (2.6) vs. 165 (2.8)	1.00 (0.76; 1.31)	0.985
4 years	202 (3.4) vs. 219 (3.7)	0.98 (0.78; 1.22)	0.866

PIM exposure was defined according to “The EURO-FORTA (Fit fOR The Aged) List Version 2”

All associations shown are not statistically significant (*p* ≥ 0.05)

*AM* appropriate medication, *CI* confidence interval, *HR* hazard ratio, *PIM* potentially inappropriate medication

^a^First imputed dataset

^b^New AM and new PIM users were matched based on a propensity score (1:1 ratio), including age, sex, income, years of education, body mass index, physical activity, alcohol consumption, smoking status, number of medications, comorbidities (including arterial hypertension, diabetes, stroke, asthma, COPD, arthritis, heart failure, myocardial infarction, atrial fibrillation, acute coronary syndrome, epilepsy, depression, bipolar disorder, Parkinson’s disease, dementia, chronic pain, osteoporosis, anemia, history of cancer), fractures in last 5 years, eGFR, HDL, LDL, HbA1c, CRP, systolic blood pressure, diastolic blood pressure, and grip strength

^c^Three- and six-month mortality is not shown because the total number of events was < 100

We conducted subgroup analyses on 6-month hospitalization, because this was the shortest follow-up duration with ≥ 100 events in each subgroup. Although no associations were statistically significant in all subgroups, higher HR point estimated were observed among females, participants aged at least 65 years, with polypharmacy, and with previous PIM use (Table 3).

**Table 3:** Main analysis in subgroups: the associations of new PIM versus new AM use with 6-month all-cause hospitalisation in 1:1 propensity-score matched population stratified by age, sex, PIM use, and polypharmacy at baseline

Subgroup	*N*_total_	*N*_Event_ (%)^a^ PIM vs. AM users	HR (95% CI)^b^	*p*
Age	11,212			
60–64	5,226	92 (3.5) vs. 94 (3.6)	1.01 (0.53; 1.91)	0.984
65–72	5,986	140 (4.7) vs. 117 (3.9)	1.27 (0.81; 1.99)	0.299
Sex	11,132			
Female	6,448	125 (3.9) vs. 101 (3.1)	1.14 (0.75; 1.73)	0.547
Male	4,684	108 (4.6) vs. 107 (4.6)	0.97 (0.55; 1.70)	0.914
PIM use at baseline	11,066			
Yes	3,476	80 (4.6) vs. 52 (3.0)	1.38 (0.67; 2.85)	0.372
No	7,590	150 (4.0) vs. 152 (4.0)	1.00 (0.72; 1.38)	0.983
Polypharmacy at baseline	10,992			
Yes	4,640	89 (3.8) vs. 73 (3.1)	1.34 (0.80; 2.25)	0.255
No	6,352	143 (4.5) vs. 133 (4.2)	1.08 (0.75; 1.56)	0.671

PIM exposure was defined according to “The EURO-FORTA (Fit fOR The Aged) List Version 2”

All associations shown are not statistically significant (*p* ≥ 0.05)

*AM* appropriate medication, *CI* confidence interval, *HR* hazard ratio, *PIM* potentially inappropriate medication

^a^First imputed dataset

^b^New AM and new PIM users were matched based on a propensity score (1:1 ratio), including age, sex, income, years of education, physical activity, body-mass-index, alcohol consumption, smoking status, number of medications, comorbidities (including arterial hypertension, diabetes, stroke, asthma, COPD, arthritis, heart failure, myocardial infarction, atrial fibrillation, acute coronary syndrome, epilepsy, depression, bipolar disorder, Parkinson’s disease, dementia, chronic pain, osteoporosis, anemia, history of cancer), fractures in last 5 years, overall health ranking, eGFR, HDL, LDL, HbA1c, CRP, systolic blood pressure, diastolic blood pressure, and grip strength

### Bias demonstration analysis I: new user design with propensity score adjustment instead of matching by indication

The associations of new PIM versus new AM use with the study outcomes are shown in Table 4. Within one month after cohort entry, 308 participants (0.8%) were hospitalized, and 326 patients (0.9%) died during the 1-year follow-up period.

**Table 4:** Bias demonstration analysis I: the associations of new PIM (*n* = 8,243) versus new AM (*n* = 28,111) use with hospitalization and all-cause mortality (*n* = 36,354) without propensity score matching per indication

Outcome	*N*_Event_ (%)^a^	Model 1^b^	Model 2^c^	p
	PIM users vs. AM users	HR (95% CI)	HR (95% CI)	
Hospitalization				
1 month	82 (1.0) vs. 226 (0.8)	1.28 (1.00; 1.65)	1.24 (0.95; 1.61)	0.107
3 months	145 (1.8) vs. 440 (1.6)	1.15 (0.95; 1.40)	1.15 (0.95; 1.39)	0.156
6 months	347 (4.2) vs. 1097 (3.9)	1.11 (0.98 1.25)	1.08 (0.95; 1.22)	0.222
1 year	589 (7.1) vs. 1976 (7.0)	1.04 (0.95; 1.14)	1.02 (0.92; 1.12)	0.739
2 years	1,049 (12.7) vs. 3487 (12.4)	1.04 (0.97; 1.12)	1.02 (0.95; 1.09)	0.672
3 years	1,572 (19.1) vs. 5,092 (18.1)	**1.07 (1.01 1.13)**	1.05 (0.99; 1.11)	0.106
4 years	2,053 (24.9) vs. 6,843 (24.3)	1.04 (0.99; 1.09)	1.02 (0.97; 1.07)	0.479
Allcause mortality^d^				
6 months	53 (0.6) vs. 115 (0.4)	**1.76 (1.27; 2.43)**	**1.55 (1.11; 2.17)**	**0.011**
1 year	104 (1.3) vs. 222 (0.8)	**1.77 (1.40; 2.24)**	**1.57 (1.24; 2.00)**	**< 0.001**
2 years	163 (2.0) vs. 438 (1.6)	**1.39 (1.16; 1.67)**	**1.27 (1.05; 1.52)**	**0.013**
3 years	224 (2.7) vs. 630 (2.2)	**1.32 (1.13; 1.54)**	**1.21 (1.04; 1.42)**	**0.015**
4 years	281 (3.4) vs. 870 (3.1)	**1.20 (1.05; 1.37)**	1.08 (0.95; 1.25)	0.225

PIM exposure was defined according to “The EURO-FORTA (Fit fOR The Aged) List Version 2”

Values in bold are statistically significant (*p* < 0.05)

*AM* appropriate medication, *CI* confidence interval, *HR* hazard ratio, *PIM* potentially inappropriate medication

^a^First imputed dataset

^b^Adjusted for age and sex

^c^Adjusted for a propensity score, including age, sex, income, years of education, body mass index, physical activity, alcohol consumption, smoking status, number of medications, comorbidities (including arterial hypertension, diabetes, stroke, asthma, COPD, arthritis, heart failure, myocardial infarction, atrial fibrillation, acute coronary syndrome, epilepsy, depression, bipolar disorder, Parkinson’s disease, dementia, chronic pain, osteoporosis, anaemia, history of cancer), fractures in last 5 years, overall health ranking, eGFR, HDL, LDL, HbA1c, CRP, systolic blood pressure, diastolic blood pressure, and grip strength

^d^Three-month mortality is not shown because the total number of events was < 100

In the age and sex adjusted model, the risk for hospitalization was non-significantly increased by 28% (HR [95% CI] 1.28 [1.00–1.65]) at one month after the index prescription. The hospitalization risk attenuated with longer follow-up time. After propensity score adjustment, the point estimates remained similar for 1-month hospitalization (HR [95% CI] 1.24 [0.95–1.61]) and the longer follow-up periods.

Moreover, we observed a significant 77% increased risk for 1-year all-cause mortality (HR [95% CI] 1.77 [1.40–2.24]) in the age and sex adjusted model. After propensity score adjustment, the risk was still significantly increased by 56% (HR [95% CI] 1.57 [1.24–2.00]). The risk attenuated over time: at 4-year follow-up, no significant association was observed.

### Bias demonstration analysis II: prevalent user design

The results for the associations of prevalent PIM use compared to non-use at the cohort’s baseline visit with the study outcomes are presented in Table 5. Within 1 month after baseline, 644 (0.3%) patients got hospitalized, and 635 (0.3%) patients deceased during the 1-year follow-up period.

**Table 5:** Bias demonstration analysis II: the associations of prevalent PIM use (*n* = 43,307) versus non-use (*n* = 173,804) with hospitalization and all-cause mortality (*n* = 217,111)

Outcome	*N*_Event_ (%)^a^	Model 1^b^	Model 2^c^	p
	PIM users vs. non-users	HR (95% CI)	HR (95% CI)	
Hospitalization				
1 month	137 (0.3) vs. 507 (0.3)	1.09 (0.90; 1.31)	1.04 (0.83; 1.31)	0.736
3 months	285 (0.7) vs. 1041 (0.6)	1.10 (0.97; 1.26)	1.01 (0.86; 1.19)	0.879
6 months	949 (2.2) vs. 3373 (1.9)	**1.13 (1.05 1.21)**	0.94 (0.86 1.03)	0.184
1 year	2,044 (4.7) vs. 7,265 (4.2)	**1.13 (1.07; 1.18)**	0.95 (0.89; 1.00)	0.071
2 years	4,252 (9.8) vs. 15,204 (8.7)	**1.12 (1.09; 1.16)**	**0.94 (0.90; 0.98)**	**0.002**
3 years	6,550 (15.1) vs. 23,593 (13.6)	**1.12 (1.09 1.15)**	**0.93 (0.90; 0.97)**	**< 0.001**
4 years	8,975 (20.7) vs. 32,673 (18.8)	**1.11 (1.09; 1.14)**	**0.94 (0.91; 0.97)**	**< 0.001**
Allcause mortality^d^				
6 months	91 (0.2) vs. 161 (0.1)	**2.15 (1.66; 2.78)**	0.96 (0.69; 1.32)	0.791
1 year	219 (0.5) vs. 416 (0.2)	**2.00 (1.69; 2.35)**	1.01 (0.83;1.24)	0.895
2 years	531 (1.2) vs. 1119 (0.6)	**1.80 (1.62; 2.00)**	0.92 (0.81; 1.05)	0.219
3 years	919 (2.1) vs. 2000 (1.2)	**1.75 (1.62; 1.89)**	0.92 (0.83; 1.01)	0.085
4 years	1,342 (3.1) vs. 3008 (1.7)	**1.70 (1.59; 1.81)**	**0.92 (0.85; 1.00)**	**0.039**

PIM exposure was defined according to “The EURO-FORTA (Fit fOR The Aged) List Version 2”

Values in bold are statistically significant (*p* < 0.05)

*CI* confidence interval, *HR* hazard ratio, *PIM* potentially inappropriate medication

^a^First imputed dataset

^b^Adjusted for age and sex

^c^Adjusted for age, sex, income, years of education, body mass index, physical activity, alcohol consumption, smoking status, number of medications, comorbidities (including arterial hypertension, diabetes, stroke, asthma, COPD, arthritis, heart failure, myocardial infarction, atrial fibrillation, acute coronary syndrome, epilepsy, depression, bipolar disorder, Parkinson’s disease, dementia, chronic pain, osteoporosis, anaemia, history of cancer), fractures in last 5 years, overall health ranking, eGFR, HDL, LDL, HbA1c, CRP, systolic blood pressure, diastolic blood pressure, and grip strength

^d^Three-month mortality are not shown because the total number of events was < 100

A 9–13% increased hospitalization risk was observed in the age and sex adjusted model and there was no clear trend with increasing follow-up time from 1 month to 4 years. With increasing case numbers, the HRs got statistically significant from the 6-month follow-up on. Furthermore, we observed a significantly, more than 2-fold increased risk for 6-month mortality in the age and sex adjusted model (HR [95% CI] 2.15 [1.66–2.78]), which decreased over the 4-year follow-up period but remained statistically significant. However, the associations between PIM use and both outcomes diminished after full adjustment, with point estimates close to one (1-month hospitalization: HR [95% CI] 1.04 [0.83–1.31]; 1-year mortality: HR [95% CI] 1.01 [0.83–1.24]). At very long follow-up times, the associations even got reversed.

Including 17,096 additional PIM users with chronic NSAIDs use in a sensitivity analysis (Table S6 (Suppl. Material)) did not alter the findings of the fully adjusted model (HR [95% CI] for 1-month hospitalization: 1.06 [0.86–1.31]; HR [95% CI] for 1-year mortality: 1.00 [0.82–1.22]).

## Discussion

### Summary of findings

This large-scale study of 11,812 1:1 propensity score matched new PIM and new AM users aged 60–69 from the UK Biobank indicated that new PIM might be associated with a 20% increased risk for 1-month hospitalization (HR, hazard ratio [95% CI, 95% confidence interval]: 1.20 [0.76–1.90) and a 23% increased risk for 1-year all-cause mortality (HR [95% CI] 1.23 [0.80–1.89]). However, the results for both outcomes were not statistically significant.

Bias demonstration analyses with 36,354 and 217,111 individuals, respectively, demonstrated the impact of common methodological challenges in pharmacoepidemiologic research on PIM: a new user design with adjustment for one common propensity score instead of matching by multiple indication specific propensity scores (*bias demonstration analysis I*) resulted in a marginally overestimated risk for 1-month hospitalization (HR [95% CI] 1.24 [0.95–1.61]) and a substantially overestimated risk for 1-year all-cause mortality (HR [95% CI] 1.57 [1.24–2.00]). Conversely, the prevalent user design (*bias demonstration analysis II*) led to null-findings for both outcomes (HRs [95% CI s]: 1-month hospitalization: 1.04 [0.83–1.31]; 1-year mortality: 1.01 [0.82–1.23]).

### Comparison with previous studies including a bias assessment

This is the first study using the new user design with active comparator propensity score matching per indication. Thus, we cannot directly compare the results of our main analysis from the UK Biobank with results from other studies. Whilst most previous studies utilized odds ratios or risk ratios to report their findings, the hazard ratios from our analyses cannot be directly compared to their results. Nevertheless, the comparison of our main analysis with our bias demonstration analyses can inform about the magnitude of over- or underestimation of the PIM risk in previous studies. Therefore, it is interesting to note that compared to our main analysis, the meta-analyses by Xing et al. [12] and Zhou et al. [13], the most recent meta-analyses for PIM use in patients aged 60 and above, reported statistically significant associations. Specifically, they yielded a 27% increased risk for hospitalization (14 cohort studies including n_total_=288,835 participants; odds ratio (OR) [95% confidence interval (CI)]: 1.27 [1.20–1.35]) [12] and 28% increase risk of mortality (44 cohort studies with n_total_=2,191,651 participants; OR 1.28 [1.20–1.36]) [13], respectively. The meta-analyses pooled studies that might have either over- or underestimated the risk due to the absence of appropriate comparators and time-dependent biases. It seems that this averaged out the bias quite well, because the pooled effect estimate is not far from the effect estimate of our main analysis.

The effect of time-dependent biases has been explored by a previous meta-analysis by Muhlack et al., that revealed a statistically significant 1.6-fold increased risk for mortality among three studies [34–36] with a new user design (risk ratio (RR) [95% CI] 1.59 [1.45–1.75]) [14]. However, the studies included in this meta-analysis did not select appropriate active comparators. Thus, this meta-analysis resembles our *bias demonstration analysis I* with a quite similar result (1-year mortality in UK Biobank: HR [95% CI] 1.57 [1.24–2.00]). In detail, the meta-analysis included a retrospective database study of 1,807,404 veterans aged 65 years and older, that reported a 1.6-fold increased odds for 1-year mortality (OR [95% CI] 1.62 [1.56–1.68]) among individuals with new PIM prescriptions compared to those without [35] and two studies involving nursing home residents aged 65 years or older [34, 36]. Dedhiya et al. [36] selected new non-PIM users as comparators, and their finding on 1-year mortality (OR [95% CI] 1.46 [1.31–1.62]) was close to the result from our *bias demonstration analysis I.* Conversely, Lau et al. [34] compared them to non-users, and the association with mortality within the same or the following month was less pronounced (OR [95% CI] 1.28 (1.05–1.55]). However, their results for one-month hospitalization (OR [95% CI] 1.27 [1.09–1.47]) are consistent with our results from the *bias demonstration analysis I* (HR [95% CI] 1.24 [0.95–1.61).

The main detriment of our *bias demonstration analyses I* and studies who followed this approach is that inappropriate comparators facilitate unmeasured confounding that can only be handled by the correct study design but not by statistical adjustment [37–40]. Thus, propensity score adjustments irrespective of the PIM indications is insufficient since the PIM indications have different underlying risks for hospitalization and mortality. By comparing PIM and AM from different indications, it is like comparing apples and oranges. Furthermore, only PIM and AM users of six different indications could not be matched because their propensity scores did not overlap or their baseline characteristics were severely imbalanced (Table S3 (Suppl. Material)). Hence, the main analysis did not include quite rare indications like dementia with safety concerning PIM classes like antipsychotics and benzodiazepines. On the other hand, quite frequent indications, like hypertension and COPD, with less safety concerning PIM, like ß-blockers and inhalative corticosteroids, were included. Thus, the strong associations between PIM and clinical outcomes reported by previous studies might result from residual confounding where they relate more to user risk profiles than to the medications themselves. This unresolved confounding may lead to an overestimation of effects, as shown in our comparison of the *bias demonstration analyses I* with the main analysis. Our main analysis addressed this methodological pitfall by a comprehensive active comparator propensity score matching per indication.

Most previous studies assessed prevalent PIM use compared to non-use and reported no significant association with mortality [14, 41–50] and hospitalization [43, 48–51]. Our *bias demonstration analysis II* replicated this approach, and produced consistent results (1-year mortality: HR [95% CI] 1.01 [0.82–1.23]; 1-month hospitalization: HR [95% CI] 1.04 [0.83–1.31]), that were further supported by the meta-analysis by Muhlack et al. of *n* = 10 trials with a prevalent user design for the outcome mortality (RR [95% CI] 1.01 [0.97–1.04]) [14]. This is not surprising because time-dependent biases, such as healthy adherer bias [29], immortal time bias [52, 53], and depletion of the susceptibles [54], may cause an underestimation of risk. Falsely strong associations can still be observed if the statistical models are not sufficiently adjusted: the 2-fold increased risk for 1-year mortality (HR [95% CI] 2.00 [1.69–2.35]) in our age and sex adjusted model, that contrasted sharply with the null result after full adjustment (HR [95% CI] 1.01 [0.82–1.23]), underscores the potential misjudgement. This may explain the strong associations between prevalent PIM use and all-cause mortality (OR [95% CI] 2.34 [1.61–3.40]) observed in a cohort study of 1117 long-term residents in Georgian nursing homes [55], since they did not adjust for key risk factors like smoking and disease severity.

Some studies reported results that were less in line with our *bias demonstration analyses.* These studies exhibited further methodological challenges, and their effects were hard to disentangle. Specifically, studies with longitudinal medication assessment that included both prevalent and new users [56–58] included immortal time biases that our *bias demonstration analysis I* avoided. Furthermore, we observed misalignment between eligibility and assignment to the study arm by using future information [35, 57, 59], which violates key principles of pharmacoepidemiology and may cause immortal time bias, selection bias, and unpredictable effects [39, 60, 61].

### Association of new PIM with early and later hospitalization and mortality risks

Whilst the point estimates of the main analysis indicated statistically non-significantly increased early hospitalization and mortality risks after new PIM prescription, we observed no association with long-term follow-ups (HRs [95% CI] 1-year hospitalization: 1.03 [0.87–1.22]; 2-year mortality: 1.00 [0.72–1.39]). This reflects the time-varying risks for adverse drug events, which typically peak during the early therapy period and decrease later on, often also due to withdrawn PIM after the adverse event got treated. Subsequently, the number of person-years at risk for both outcomes does not equal the number of person-years of PIM exposure [54, 62]. This underscores the necessity of short-time follow-ups when assessing PIM safety, even if the limited number of events may affect the statistical power.

### Subgroup analyses

When we conducted our main analysis stratified by subgroups, higher HR point estimates were observed among females, participants aged at least 65 years, with polypharmacy, and with previous PIM use. Given that these are known risk factors for adverse drug events [63, 64] and drug-related hospitalizations [65], the stronger effect estimates in these subgroups are plausible. These results underscore the importance of careful prescribing practices and thorough checks for potential drug interactions when prescribing new medications in these particularly vulnerable populations.

Our subgroup analysis of new PIM users who had at least one different PIM use at baseline suggested a stronger association with 6-month hospitalization (HR [95% CI] 1.38 [0.67–2.85]) than in the subgroup of study participants with no PIM exposure at baseline (HR [95% CI] 1.00 [0.72–1.38]). This finding is supported by the prospective cohort study of Weir et al., who observed that the risk for a combined endpoint of emergency department visits, rehospitalizations, and death within 30 days after hospital discharge increased by 13% (HR [95% CI] 1.13 (1.03–1.26)) per each newly prescribed PIM and only by 5% (HR [95% CI] 1.05 [1.01–1.10]) per each new non-PIM [66].

Similarly, our subgroup analysis suggested that new PIM users with polypharmacy at baseline had a higher risk for 6-month hospitalization (HR [95% CI] 1.34 [0.80–2.25]) than those without polypharmacy (HR [95% CI] 1.08 [0.75–1.56]). This may be explained by the pharmacologic profile of many PIMs, that are known for their potential to cause drug-drug interactions [67]. Consequently, the risk for drug-drug interactions increases with the number of comedications, and it is particularly high among participants exposed to polypharmacy.

Furthermore, we observed a non-significantly 23% increased risk for 6-month hospitalization in our subgroup analysis of participants aged 65–72 years (HR [95% CI] 1.27 [0.81–1.99]), but no increased risk in those aged 60–64 (HR [95% CI] 1.01 [0.53–1.91]), which supports the widely used cut-off of 65 years for defining PIM. An older age of a study population may generally lead to the observations of stronger associations of PIM with hospitalizations, since studies involving very old participants with mean ages of 73–80 years reported much higher hospitalization risks than we observed in our study with a mean age of 64 years [41, 46, 57], which is a relatively young study population for PIM research. However, the results are not directly comparable due to the different methodological approaches.

A potentially higher risk for 6-month hospitalization among females (HR [95% CI] 1.14 [0.75–1.73]) compared to males (HR [95% CI] 0.97 [0.55; 1.70]) might be attributed to their higher susceptibility to adverse drug reactions, including severe events such as cardiac arrythmias, and the underrepresentation of females in clinical trials [68, 69].

### Strengths and limitations

This is not only one of the largest studies assessing the association between PIM and clinical outcomes in the general older population, it is also the first study applying a new user/active comparator approach, the gold standard in pharmacoepidemiology that minimizes typical biases and unmeasured confounding [37, 60, 70]. Nonetheless, it is important to underscore that unmeasured confounding can never be entirely eliminated. However, this gold standard approach also has the major limitation that the propensity score matching by indication substantially reduced the study population and subsequently the statistical power. Since primary care linkage was solely available for approximately half of the cohort, this unfortunately led to non-statistically significant results in the main analysis. An even larger sample size than the *n* = 95,187 UK Biobank participants with longitudinal medication assessment will be needed to verify the approximately 20% increased risk for hospitalization and mortality among new PIM users. However, we are confident that such studies will be conducted in future with large claims data sets who include millions of study participants (e.g. Medicare data from the US or the Clinical Practice Research Datalink (CPRD) from the UK). Such larger studies could also explore cause-specific mortality, which was not possible in our data set due to case number restrictions, and distinguish between unplanned and elective hospitalizations.

A strength of using the UK Biobank and not claims data is the available of important confounders usually not available in claims data sets, such as life-style factors. However, our data set al.so had limitations. We cannot rule out misclassification due to non-adherence because this was not assessed. Furthermore, the data did not allow to account for the treatment durations and changes within the two-year follow-up period. We addressed these limitations by applying an intention-to-treat principle with short-term follow-ups as primary outcomes. Furthermore, the healthy volunteer bias of the UK Biobank population [71] and the relatively young age of the participants are limitations. The UK Biobank mainly recruited participants up to the age of 69 years. Thus our results cannot be generalized to older populations. As particularly the oldest age groups have an increased risk for adverse outcomes by PIM and approx. half of our study population was even younger than 65 years, it is likely that future studies with older study populations will obtain stronger effect estimates for the risks of PIM use with hospitalizations and mortality. Generalizability to other countries may also be limited because the prescribing patterns of physicians vary largely in different health care systems. Lastly, it should be noted that PIM lists, including the EURO-FORTA list, are expert consensus-based, which resembles low evidence in the evidence-based medicine hierarchy.

### Implications for bias assessment guidelines in pharmacoepidemiology

Interestingly, recent systematic reviews about PIM use judged 39 out of 44 studies on all-cause mortality [13] and 12 out of 16 studies on hospitalization [12] as having a low risk of bias according to the Newcastle Ottawa Scale. Although reporting guidelines for pharmacoepidemiology are now available [22, 72], current bias assessment tools do not sufficiently cover the specific methodological challenges of pharmacoepidemiologic research. Given that these challenges have been insufficiently recognized in previous research, we promote the development of tailored bias assessment tools to enhance future quality of studies on this research topic.

## Conclusion

This first study utilizing the gold standard methodology demonstrated that all previous studies on PIM have either over- or underestimated the risks for hospitalizations and all-cause mortality by overlooking typical pitfalls of pharmacoepidemiology. Future research should overcome these limitations by using the new user/active comparator approach but will need large data sets, preferably including older individuals, to detect moderately increased risks of approximately 20% with statistical significance.

## Supplementary Information

Below is the link to the electronic supplementary material.

Supplementary Material (DOCX 727 KB)

## Data Availability

The study used data from the UK Biobank. Publicly available data is accessible to researchers via an open application on https://www.ukbiobank.ac.uk/register-apply/.
