# Supplementary material for: Associations of potentially inappropriate medications in older adults with mortality and hospitalizations: methodological challenges in pharmacoepidemiology
Source: Eur J Epidemiol. 2025 Aug 23;40(9):1067–82. doi: 10.1007/s10654-025-01294-x (PMC12537759; doi:10.1007/s10654-025-01294-x)
Supplement: Supplementary file 1 — Supplementary Material (DOCX 727 KB) [file 10654_2025_1294_MOESM1_ESM.docx]

**Supplementary Material to Article**

“Methodological challenges in pharmacoepidemiologic studies aiming to provide evidence for hospitalization and mortality rate reductions by avoiding potentially inappropriate medication in older adults. A UK Biobank analysis”

Table of contents

[Table S1. EURO-FORTA classification and ATC codes of the assessed medications 2](#_Toc201827111)

[Methods S1. Assignment of medications to FORTA indications 10](#_Toc201827112)

[Table S2. Percentages of missing data of covariates prior to imputation in population analyzed (N=217,111) 11](#_Toc201827113)

[Methods S2. Propensity score matching used for main analysis 13](#_Toc201827114)

[Table S3. Overview of 1:1 propensity score matching of new PIM and new AM users per indication (N_total_=36,534) 14](#_Toc201827115)

[Figure S1. Distribution of propensity scores for receiving new PIM versus new AM within two years after baseline by quintile and treatment (N=36,534) in 1^st^ and 2^nd^ imputed dataset 15](#_Toc201827116)

[Figure S2. Distribution of propensity scores for receiving new PIM versus new AM within two years after baseline by quintile and treatment (N=36,534) in 3^rd^ and 4^th^ imputed dataset 16](#_Toc201827117)

[Figure S3. Distribution of propensity scores for receiving new PIM versus new AM within two years after baseline by quintile and treatment (N=36,534) in 5^th^ and 6^th^ imputed dataset 17](#_Toc201827118)

[Figure S4. Distribution of propensity scores for receiving new PIM versus new AM within two years after baseline by quintile and treatment (N=36,534) in 7^th^ and 8^th^ imputed dataset 18](#_Toc201827119)

[Figure S5. Distribution of propensity scores for receiving new PIM versus new AM within two years after baseline by quintile and treatment (N=36,534) in 9^th^ and 10^th^ imputed dataset 19](#_Toc201827120)

[Table S4. Assessment of the balance of baseline covariates between new PIM and new AM users after propensity score adjustment (N=36,354) 20](#_Toc201827121)

[Table S5. Baseline characteristics of propensity score matched new PIM and AM users 22](#_Toc201827122)

[Table S6. The associations of prevalent PIM use including chronic use of NSAIDs (n=60,403) versus non-use (n=156,708) with hospitalization and all-cause mortality (n=217,111) 25](#_Toc201827123)

# Table S1. EURO-FORTA classification and ATC codes of the assessed medications

| **Medication** | **Class** | **ATC codes** |
| --- | --- | --- |
| ***Arterial hypertension*** |  |  |
| ACE-inhibitors,  angiotensin receptor antagonists | A | C09A, C09B, C09C, C09DA, C09DB, C10BX04, C10BX06, C10BX07, C10BX11, C10BX12, C10BX14, C10BX15, C10BX17, C10BX18, C10BX21, C09DX01, C09DX03, C09DX06, C09DX07, C09DX08, C10BX10, C10BX16, C10BX19, C10BX20 |
| Long-acting calcium antagonists, dihydropyridine type | A | C08CA, C08GA, C09DB, C09BB, CO7FB, C10BX03, C10BX07, C10BX09, C10BX11, C10BX18, C10BX19, C09DX01, C09DX03, C09DX06, C09DX07, C09DX08, |
| Indapamide | A | C03BA11, C09BX01, C09BX06, C10BX13 |
| Other diuretics | B | C03A, C03B, C03C, C03E, C03DB, C09BA, C09DX01, C09DX03, C09DX06, C09DX07, C09DX08, C09BX01, C09BX03, C09BX06, C10BX13 |
| Alpha receptor antagonists | C | C02CA; C02LE |
| Spironolactone | C | C03DA01, C03EA13, C03EA07, C03EA01, C03EA02 |
| Moxonidine | C | C02AC05, C02LC05 |
| Aliskiren | C | C09XA02, C09XA53, C09XA52, C09XA54, C09DX02 |
| Betablockers^a^ | C | C07A |
| Atenolol^a^ | D | C07AB03, C07AB11 |
| Clonidin | D | C02AC01, C02LC01,C02LC51 |
| Minoxidil | D | C02DC01 |
| Calcium antagonists, verapamil type | D | C08D, C09BB10 |
| ***Cardiac insufficiency*** |  |  |
| ACE-inhibitors, angiotensin receptor antagonists | A | C10BX04, C10BX06, C10BX07, C10BX11, C10BX12, C10BX14, C10BX15, C10BX17, C10BX18, C10BX21, C09DX01, C09DX03, C09DX06, C09DX07, C09DX08, C10BX10, C10BX16, C10BX19, C10BX20, C09DX04 |
| Betablockers | A | C07A; excluding (C07AA07), pindolol (C07AA03), propranolol (C07AA05), and carteolol (C07AA15) |
| Diuretics | A | C03A, C03B, C03C, C03E, C03DB, C09BA, C09DX01, C09DX03, C09DX06, C09DX07, C09DX08, C09BX01, C09BX03, C09BX06, C10BX13 |
| SGLT2 inhibitors | B | A10BK, A10BD15, A10BD16, A10BD19, A10BD20, A10BD21, A10BD23, A10BD24, A10BD25, A10BD27, A10BD29 |
| Spironolactone | B | C03DA01, C03EA13, C03EA07, C03EA01, C03EA02 |
| Digitalis preparations | C | C01AA, C01AA02, C01AA52, C01AA08, C01AA04, C01AA01 |
| Ivabradine | C | C01EB17, C07FX06, C07FX05 |
| ***Acute coronary syndromes*** |  |  |
| ACE-inhibitors, angiotensin receptor antagonists | A | C09A, C09B, C09C, C09DA, C09DB, C10BX04, C10BX06, C10BX07, C10BX11, C10BX12, C10BX14, C10BX15, C10BX17, C10BX18, C10BX21, C09DX01, C09DX03, C09DX06, C09DX07, C09DX08, C10BX10, C10BX16, C10BX19, C10BX20 |
| Low-dose ASA | A | B01AC06, C10BX08, C10BX12, C10BX06, C07FX04, C07FX03, C10BX02, C10BX05, C10BX01, C10BX04, C07FX02, B01AF51 |
| Heparin | A | B01AB |
| Betablockers^b^ | A | C07A; excluding sotalol (C07AA07) |
| Atorvastatin | A | C10AA05, C10BX08, C10BX03, C10BA05, C10BA08, C10BX15, C10BX12, C10BX06, C10BX19, C10BX11, C10BX18 |
| Nitroglycerin spray, on demand | A | C01DA02^c^ |
| Clopidogrel, prasugrel^d^ | B | B01AC04, B01AC22 |
| Thrombolytics, especially rTPA | B | B01AD |
| Nitrates, long term | C | C01DA02,^e^ C01DA08,^f^ C01DA14, C01DA05 |
| Glycoprotein IIb/IIIa antagonist | C | B01AC13, B01AC16, B01AC17 |
| Ivabradine | C | C01EB17, C07FX06, C07FX05 |
| ***Myocardial infarction*** |  |  |
| ACE-inhibitors, angiotensin receptor antagonists | A | C09A, C09B, C09C, C09DA, C09DB, C10BX04, C10BX06, C10BX07, C10BX11, C10BX12, C10BX14, C10BX15, C10BX17, C10BX18, C10BX21, C09DX01, C09DX03, C09DX06, C09DX07, C09DX08, C10BX10, C10BX16, C10BX19, C10BX20 |
| Low dose ASA | A | B01AC06, C10BX08, C10BX12, C10BX06, C07FX04, C07FX03, C10BX02, C10BX05, C10BX01, C10BX04, C07FX02, B01AF51 |
| Clopidogrel^g, h^ | A | B01AC04 |
| Nitroglycerin spray, on demand | A | C01DA02^c^ |
| Statins | A, B^i^ | C10AA, C10BA, C10BX |
| Betablockers | A,^b^ C^j^ | C07; excluding sotalol (C07AA07) |
| Nitrate, long-term use | C | C01DA02,^e^ C01DA08,^f^ C01DA14, C01DA05 |
| Ezetimibe | C | C10AX09, C10BA05, C10BA10, C10BA11, C10BA12, C10BA06, C10BA02 |
| Fibrates | C | C10AB |
| Amiodarone | C | C01BD01 |
| Other class-I/III antiarrhythmic agents | D | C01BA, C01BB, C01BC, C01BG, C01BD02, C01BD03, C01BD04, C01BD05, C01BD06, C01BD07, C07AA07, C07FX02, C07BA07 |
| Dihydropyridine antagonists^k^ | D | C08CA, C08GA, C10BX03, C10BX09, C10BX11, C10BX14, C10BX18, C10BX19 |
| Niacin | D | C10AD |
| ***Stroke*** |  |  |
| Low dose ASA | A | B01AC06, C10BX08, C10BX12, C10BX06, C07FX04, C07FX03, C10BX02, C10BX05, C10BX01, C10BX04, C07FX02, B01AF51 |
| Statins | A | C10AA, C10BA, C10BX |
| r-TPA | A | B01AD |
| Clopidogrel | A | B01AC04 |
| Anticoagulants, including NOACs | A | B01AA, B01AE, B01AF |
| Dipyridamole plus ASA | B | B01AC07 |
| ***Atrial fibrillation*** |  |  |
| Betablockers | A | C07; excluding sotalol (C07AA07) |
| Oral vitamin K antagonists | B | B01AA03 |
| Digoxin | B | C01AA05 |
| NOACs except Dabigatran | B | B01AF |
| Dabigatran | C | B01AE07 |
| Heparin^l^ | C | B01AB |
| Digitoxin | C | C01AA04 |
| Diltiazem, verapamil | C | C08DB01, C08DA01, C09BB10, C08DA51 |
| Amiodarone | C | C01BD01 |
| Other class I or III antiarrhythmic agents | D | C01BA, C01BB, C01BC, C01BG, C01BD02, C01BD03, C01BD04, C01BD05, C01BD06, C01BD07, C07AA07, C07FX02, C07BA07 |
| Low dose ASA^m^ | D | B01AC06, B01AC56, C10BX08, C10BX12, C10BX06, C07FX04, C07FX03, C10BX02, C10BX05, C10BX01, C10BX04, C07FX02 |
| ***COPD*** |  |  |
| Inhalative long-acting parasympatholytics | A | R03BB02, R03BB03, R03BB04, R03BB05, R03BB06, R03BB07, R03BB54, R03AL03, R03AL04, R03AL05, R03AL06 |
| Antibiotics except fluoroquinolones | A | J01A, J01B, J01C, J01D, J01F, J01G, J01XA, J01XB, J01XC, J01XD, J01XX02, J01XX03, J01XX04, J01XX05, J01XX06, J01XX08, J01XX09, J01XX10, J01XX11, J01XX12 |
| Inhalative beta 2 mimetic agents | B | R03AC |
| Systemic glucocorticoids | A, D^n^ | H02 |
| Inhalative glucocorticoids^o^ | C | R03BA, R03AK06, R03AK07, R03AK08, R03AK09, R03AK10, R03AK11, R03AK12, R03AK13, R03AK14, R03AL08, R03AL09, R03AL11, R03AL12 |
| Mucolytic agents | C | R05CB |
| Roflumilast | C | R03DX07 |
| Theophylline | D | R03DA04, R03DB04, R03DA54, R03DA74 |
| Antitussives | D | R05DA, R05DB |
| ***Osteoporosis*** |  |  |
| Calcium and vitamin D | A | A12A, A11CC05, A11CC55 |
| Bisphosphonates | A, B^p^ | M05BA |
| Raloxifene | B | G03XC01 |
| Denosumab | B | M05BX04 |
| Alfacalcidol | C | M05BB06, A11CC03, A11CC04 |
| Parathormone, teriparatide | C | H05AA |
| Strontium ranelate | D | M05BX03, M05BX53 |
| Nandrolone decanoate | D | A14AB01 |
| Fluoride | D | A12CD |
| Hormone replacement therapy | D | G03C, G03F, G03AA, G03AB |
| ***Diabetes*** |  |  |
| Metformin | A | A10BA02, A10BD17, A10BD13, A10BD16, A10BD15, A10BD20, A10BD23, A10BD22, A10BD18, A10BD11, A10BD26, A10BD05, A10BD14, A10BD03, A10BD10, A10BD07, A10BD02, A10BD28, A10BD08, A10BD27, A10BD25 |
| DPP-4 inhibitors | B | A10BH, A10BD07, A10BD09, A10BD10, A10BD11, A10BD12, A10BD13, A10BD14, A10BD25, A10BD27, A10BD28 |
| Insulin | B | A10A |
| GLP-1-RAs | B | A10BJ |
| Acarbose | C | A10BF01, A10BD17 |
| SGLT2-inhibitors^q^ | C | A10BK, A10BD15, A10BD16, A10BD19, A10BD20, A10BD21, A10BD23, A10BD24, A10BD25, A10BD27 |
| Glinides | C | A10BX02, A10BX03, A10BX05, A10BX08, A10BD14 |
| Sulfonylureas | C, D^r^ | A10BB12, A10BB31, A10BB06, A10BB02, A10BB10, A10BB05, A10BB03, A10BB01, A10BB04, A10BB09, A10BB07, A10BB08, A10BB11, A10BD01, A10BD02, A10BD06 |
| PPARγ ligands | C, D^s^ | A10BG, A10BD09, A10BD12, A10BD04, A10BD03, A10BD05, A10BD06, A10BD26 |
| ***Dementia*** |  |  |
| Acetylcholinesterase inhibitors | B | N06DA |
| Memantine | C | N06DX01 |
| Ginkgo biloba | D | N06DA53, N06DX02, N06DX30 |
| Statins^t^ | D | C10AA, C10BX, C10BA01, C10BA02, C10BA03, C10BA04, C10BA05, C10BA06, C10BA07, C10BA08, C10BA09, C10BA11, C10BA12 |
| Piracetam | D | N06BX03 |
| Antioxidants: vitamin E, selenium, vitamin C | D | A11G, A11EB, A11HA03, A11CB |
| Ginseng | D | A13AP02 |
| Hormone preparations^u^ | D | G03 |
| Selegiline^v^ | D | N04BD01 |
| Nimodipine | D | C08CA06 |
| Ergoline derivates | D | C04AE |
| Pyritinol | D | C08CA06 |
| Antiphlogistics, e.g. indomethacin | D | M01AB01, M01AB51 |
| Desferrioxamine | D | V03AC01 |
| ***Dementia-associated depression*** |  |  |
| SSRIs | C | N06AB |
| Mirtazapine | C | N06AX11 |
| SNRIs | C | N06AX16, N06AX23, N06AX21, N06AX17, N06AX28 |
| ***Dementia-associated paranoia, hallucination*** |  |  |
| Risperidone | C | N05AX08 |
| Melperone | C | N05AD03 |
| Quetiapine | C | N05AH04 |
| Aripiprazol | C | N05AX12 |
| Haloperidol | C | N05AD01 |
| Clozapin | D | N05AH02 |
| ***Dementia-associated insomnia*** |  |  |
| Melatonin | C | N05CH01 |
| Mirtazapine | C | N06AX11 |
| Doxepin | D | N06AA12 |
| Z-drugs | C | N05CF |
| ***Dementia-associated restlessness, agitation*** |  |  |
| Trazodon |  | N06AX05 |
| Risperidone | C | N05AX08 |
| Melperone | C | N05AD03 |
| Quetiapine | C | N05AH04 |
| Citalopram^w^ | C | N06AB04, N06AB10 |
| Clomethizole | D | N05CM02 |
| Pipamperone | D | N05AD05 |
| ***Depression*** |  |  |
| SSRIs | B | N06AB |
| Tricyclic antidepressants | C | N06AA,^x^ N06CA01 |
| Mirtazapine | C | N06AX11 |
| SNRIs | C | N06AX16, N06AX23, N06AX21, N06AX17, N06AX28 |
| Bupropion | C | N06AX12 |
| Quetiapine^y^ | C | N05AH04 |
| Vortiotexine | C | N06AX26 |
| Trazodone | C | N06AX05 |
| Olanzapine | C | N05AH03 |
| Moclobemide | D | N06AG |
| Benzodiazepines^z^ | D | N05CD, N05BA, N03AE |
| St. Johns Wort | D | N06AX25 |
| Agomelatine | D | N06AX22 |
| Reboxetine | D | N06AX18 |
| ***Bipolar disorder*** |  |  |
| Lithium | B | N05AN |
| Quetiapin | B | N05AH04 |
| Valproic acid | C | N03AG01 |
| Lamotrigine^z^ | C | N03AX09 |
| Carbamazepine | D | N03AF01 |
| ***Insomnia*** |  |  |
| Melatonin^aa^ | B | N05CH01 |
| Z-drugs | C | N05CF |
| Pipamperone | C | N05AD05 |
| Melperone | C | N05AD03 |
| Mirtazapine | C | N06AX11 |
| Doxepin | D | N06AA12 |
| Benzodiazepines^z^ | D | N05CD, N05BA, N03AE |
| Opipramol | D | N06AA05 |
| Sedative antihistamines | D | R06AA02, R06AA52, R06AC01, R06AC03, R06AC53, R06AX05, R06AC04, R06AA08, R06AA09, R06AA59, R06AA01, R06AA04, R06AA54, R06AA11, R06AA61, R06AB05, R06AB04, R06AB54, R06AB02, R06AB52, R06AB06, R06AB56, R06AB01, R06AB51, R06AX07, R06AE03, R06AE53, R06AE04, N05BB01, N05BB51, R06AE05, R06AE55, R06AD02, R06AD52, R06AD01, R06AX02, N05CM27, N05CM20 |
| ***Pain*** |  |  |
| Paracetamol | A | N02BE01, N02BE51, N02AJ06, N02AJ13 |
| Opioids, e.g. Buprenorphine, oxycodone, hydromorphone, fentanyl | B | N02AB03, N02AE01, N02AA05, N02AA03 |
| Morphine | C | N02AA01, N02AA51, N02AG01 |
| Opioid agonist/antagonist combinations | C | N02AX51, N02AA55, N02AA53, N02AA56 |
| Metamizole | C | N02BB02, N02BB52 |
| Tramadol | C | N02AX02, N02AJ14, N02AJ15, N02AJ13, N02AJ16 |
| SSRIs^ab^ | C | N06AB |
| SNRIs | C | N06AX16, N06AX23, N06AX21, N06AX17, N06AX28 |
| Antiepileptics^z, ac^ | C, D^ad^ | N03AF02, N03AF04, N03AX15, N03AB02, N03AB52, N03AA02, N03AD01, N03AD51, N02BF01, N03AG01, N03AF01 |
| Tricyclic antidepressants | D | N06AA,^x^ N06CA01 |
| NSAIDs for long-term use^ae^ | D | M01AA, M01AB, M01AC, M01AD, M01AE01, M01AE02, M01AE03, M01AE04, M01AE05, M01AE06, M01AE07, M01AE08, M01AE09, M01AE10, M01AE11, M01AE12, M01AE13, M01AE14, M01AE15, M01AE16, M01AE17, N02BA51, N02BA71, M01BA01, M01BA03, N02BA01 |
| ***Epilepsy*** |  |  |
| Levetiracetam | B | N03AX14 |
| Lamotrigine | B | N03AX09 |
| Gabapentin | B | N02BF01 |
| Topiramat | B | N03AX11 |
| Lorazepam | B, D^af^ | N05BA06, N05BA56 |
| Pregabalin | C | N02BF02 |
| Valproic acid | C | N03AG01 |
| Carbamazepine | C | N03AF01 |
| Eslicarbamazepine | C | N03AF04 |
| Oxcarbamazepine | C | N03AF02 |
| Zonisamide | C | N03AX15 |
| Lacosamide | C | N03AX18 |
| Diazepam | C, D^ag^ | N05BA01 |
| Midazolam | C, D^ag^ | N05CD08 |
| Phenytoin | D | N03AB02, N03AB52 |
| Phenobarbital | D | N03AA02 |
| Ethosuximide | D | N03AD01, N03AD51 |
| ***Parkinsons disease*** |  |  |
| Levodopa | A | N04BA01, N04BA02, N04BA03 |
| COMT inhibitors | B | N04BX02, N04BX04, N04BA03 |
| Ropinirole | B | N04BC04 |
| Piribedil | B | N04BC08 |
| Quinagolide | B | G02CB04 |
| Rotigotine | B | N04BC09 |
| Pramipexole | C | N04BC05 |
| MAO-B inhibitors | C | N04BD02, N04BD01, N04BD03 |
| Bromocriptine | D | N04BC01 |
| Cabergoline | D | N04BC06 |
| Amantadine | D | N04BB01 |
| Anticholinergics | D | N04A |
| ***Incontinence*** |  |  |
| Fesoterodine | B | G04BD11 |
| Tolterodine | C | G04BD07 |
| Trospium chloride | C | G04BD09 |
| Oxybutynin | C, D^ah^ | G04BD04 |
| ***Gastrointestinal illness*** |  |  |
| Proton pump inhibitors | B | A02BC |
| H_2_ receptor antagonists | C | A02BA |

Abbreviations: ASA, acetylsalicylic acid; ATC, Anatomical Therapeutic Chemical Classification; COMT, catechol-O-methyltransferase; COPD, chronic obstructive pulmonary disease; DPP-4, dipeptidyl peptidase-4; eGFR, estimated glomerular filtration rate; GLP-1-RA, Glucagon-like peptide-1 receptor agonists; MAO, monoamine oxidase; NOACs, new oral anticoagulants; NSAIDs, non-steroidal anti-inflammatory drugs; PPARγ, peroxisome proliferator-activated receptor gamma; SGLT2, sodium glucose linked transporter 2; SNRIs, serotonin-noradrenalin reuptake inhibitors; SSRIs, selective serotonin reuptake inhibitors.

^a^ In participants without diagnosis of acute coronary syndrome, myocardial infarction, atrial fibrillation, and cardiac insufficiency.
^b^ In participants with history of myocardial infarction, betablockers are only considered AM if the date of the last myocardial infarction was ≤ 3 years before the prescription.
^c^ Only considered as AM if identified as immediate-release preparation. Glyceryl trinitrate patches were not considered.
^d^ If prescribed within 1 year after diagnosis of myocardial infarction.
^e^ Patches.
^f^ Preparations with extended release.
^g^ Up to 12 months after diagnosis or in case of ASA intolerance.
^h^ Participants receiving ASA prescriptions after their clopidogrel prescription were not considered as being ASA intolerant.
^i^ In participants aged > 85 years. ^j^ Only considered as PIM if last diagnosis of myocardial infarction was > 3 years ago. Not considered as PIM in participants with cardiac insufficiency or atrial fibrillation.
^k^ Only considered as PIM in participants without arterial hypertension.
^l^ Only considered as PIM in patients without diagnosis of acute coronary syndromes.
^m^ Only considered as PIM in participants without acute coronary syndrome, cardiac insufficiency, and stroke.
^n^ A: acute, short-term use (5-7 days) in cases of exacerbation. D: chronic use. Not considered in the analyses since it was not possible to assess exacerbations and the duration of use from prescription data.
^o^ Not considered as PIM in participants with asthma.
^p^ A: preparations for intravenous use. B: preparations for oral intake.
^q^ Not considered as PIM in participants with cardiac or renal insufficiency.
^r^ 1^st^ generation sulfonylureas are classified as D.
^s^ Rosiglitazone.
^t^ Not considered as PIM in participants with acute coronary syndrome, myocardial infarction, and stroke.
^u^ Only considered as PIM in participants without osteoporosis.
^v^ In participants without Parkinson’s disease.
^w^ Including escitalopram.
^x^ Doxepin (N06AA12) only in participants without insomnia.
^y^ Only considered as PIM in participants without bipolar disorder.
^z^ Only considered as PIM in participants without epilepsy.
^aa^ In participants without dementia.
^ab^ In participants without depression.
^ac^ Valproic acid (N03AG01) and carbamazepine (N03AF01) were only considered as PIM in participants without bipolar disorder.
^ad^ C: antiepileptics excluding carbamazepine. D: carbamazepine.
^ae^ If regular use was verified through primary care prescription data or if participant reported regular use of ibuprofen or aspirin in the baseline questionnaire.
^af^ B: emergency use. D: long-term use. Not relevant since no participants with epilepsy and lorazepam use were identified.
^ag^ C: emergency use. D: long-term use.
^ah^ C: extended release. D: immediate release.

# Methods S1. Assignment of medications to FORTA indications

In particular hypertension and other cardiovascular diseases often occur coincidently and the indicated treatments may overlap, making it difficult to assign the identified medication to one of the assessed indications. E.g., ACE inhibitors might be indicated in arterial hypertension, heart failure, acute coronary syndromes, and after myocardial infarction.

To perform the indication-wise matching of PIM and AM users, it was necessary to clearly assign the identified medication to one indication. Therefore, the following decisions were made:

1. Arterial hypertension definition: only included participants if the medication was clearly assigned to treat arterial hypertension because
   1. there was no other cardiovascular indication for the relevant drug (e.g., Aliskiren); or
   2. the patient was only diagnosed with arterial hypertension and not with other potential indications for the treatment. E.g., Ramipril prescribed for a participant that was diagnosed with arterial hypertension, but not with heart failure, acute coronary syndromes, or myocardial infarction
2. Cardiovascular diseases definition: included participants if
   1. the medication was assigned to one of the following conditions: heart failure, myocardial infarction, atrial fibrillation, acute coronary syndromes, or stroke due to the broad overlap of potential treatments and the frequent coincidence of diagnoses; or
   2. the medication is indicated to treat ~~was assigned to~~ arterial hypertension and at least one additional cardiovascular disease, as outlined in 2a), and the patient is diagnosed with multiple overlapping conditions. E.g., Ramipril prescribed in a participant diagnosed with heart failure and arterial hypertension
3. Medications to treat dementia and dementia-associated syndromes were pooled together.
4. In cases of overlap between conditions and treatments, the medication was assigned to the more relevant condition (e.g., in participants diagnosed with epilepsy and chronic pain, antiepileptics were assigned to epilepsy).

# Table S2. Percentages of missing data of covariates prior to imputation in population analyzed (N=217,111)

| **Characteristics** | **Proportion missing (%)** |
| --- | --- |
| ***Sociodemographic/ -economic factors*** | - |
| Sex | 0 |
| Age | 0 |
| Income^a^ | 19.4 |
| Years of schooling | 1.1 |
| ***Lifestyle factors*** |  |
| BMI *(kg/m^2^)* | 0.1 |
| Physical activity *(MET-min/week)* | 25.9 |
| Alcohol consumption *(g/day)* | 0.2 |
| Smoking status | 0.1 |
| Years since quit | 13.3 |
| ***Comorbidities*** | - |
| Diabetes | 0 |
| Arterial hypertension | 0 |
| Stroke | 0 |
| Myocardial infarction | 0 |
| Heart failure | 0 |
| Acute coronary syndromes | 0 |
| Aterial fibrillation | 0 |
| Asthma | 0 |
| COPD | 0 |
| Arthritis | 0 |
| Osteoporosis | 0 |
| History of fractures^b^ | 0 |
| Pain^c^ | 0 |
| Parkinson’s disease | 0 |
| Dementia | 0 |
| Depression | 0 |
| Bipolar disorder | 0 |
| Insomnia | 0 |
| Epilepsy | 0 |
| Gastrointestinal illness^d^ | 0 |
| Incontinence | 0 |
| Anaemia | 0 |
| History of cancer | 0 |
| Overall health rating^e^ | 0.5 |
| Number of medications | 0 |
| ***Functional assessments*** | - |
| Systolic blood pressure *(mmHg)* | <0.1 |
| Diastolic blood pressure *(mmHg)* | <0.1 |
| Grip strength *(kg)* | 0.1 |
| eGFR^f^ *(ml/min/1.73m²)* | 2.1 |
| ***Blood-based biomarkers*** | - |
| CRP *(mg/L)* | 2.2 |
| HDL *(mmol/L)* | 4.1 |
| LDL *(mmol/L)* | 2.2 |
| HbA_1c_ *(mmol/L)* | 2.3 |

Abbreviations: BMI, body mass index; COPD, chronic obstructive pulmonary disease; CRP, c-reactive protein; eGFR, estimated glomerular filtration rate; HbA_1c_, haemoglobin A_1c_; HDL, high-density lipoprotein; LDL, low-density lipoprotein, MET, metabolic equivalent of task.

^a^ Average total household income before tax.
^b^ Fractures in last five years.
^c^ Combination of pain-related diagnoses (including migraine, other headache syndromes, facial pain, chronic backpain, chronic pain syndromes, arthritis, osteoarthritis, urinary tract stones, and gout) and self-reported pain in the last month.
^d^ Including regular use of of non-steroidal anti-inflammatory drugs.
^e^ Self-reported.
^f^ Calculated based on CKD-EPI equation

# Methods S2. Propensity score matching used for main analysis

We applied optimal fixed ratio matching in indications with a large number of new AM users and few new PIM users and greedy nearest neighbour matching for indications with fewer new AM compared to new PIM users or few overlap in propensity scores, respectively. Caliper ≤0.2 was used and stepwise increased by 0.1 if optimal matching was not possible for an indication. To assess the balance produced by matching, we calculated standardized mean differences for all covariates, with standardized differences <0.25 considered well-balanced. An overview is provided in **Supplemental Table S2 (Suppl. Material)**.

# Table S3. Overview of 1:1 propensity score matching of new PIM and new AM users per indication (N_total_=36,534)

| **Indication** | **N_total_(N_PIM_; N_AM_)^a^** | **Matched pairs^a^** | **PS_PIM_^a,b^** | **PS_AM_^a,b^** | **Matching method^c^** | **Caliper** | **Standardized differences^d^** |
| --- | --- | --- | --- | --- | --- | --- | --- |
| 1. Arterial hypertension | 4,810 (582; 4,228) | 581 | 0.0275‑0.5063 | 0.000-0.4318 | OFR | 0.3 | **<0.25** |
| 2. Cardiovascular diseases^e^ | 4,225 (501; 3,724) | 501 | 0.0099-0.6819 | 0.0028-0.6711 | OFR | 0.3 | **<0.25** |
| 3. COPD | 5,857 (391; 5,466) | 390 | 0.0119-0.2823 | 0.0084-0.2397 | OFR | 0.1 | **<0.25** |
| 4. Osteoporosis | 740 (174; 566) | 170 | 0.0332-0.7784 | 0.0000-0.6445 | GNN^f^ | 0.3 | **<0.25** |
| 5. Diabetes | 954 (360; 594) | 244 | 0.0288-1.0000 | 0.0093-0.9213 | GNN^g^ | 0.3 | **<0.25** |
| 6. Dementia^h^ | 127 (114; 13) | 0^i^ | 0.9971-1.0000 | 0.0000-0.0025 | - | - | **-** |
| 7. Depression | 1,244 (714; 530) | 461 | 0.0989-1.0000 | 0.0738-0.9069 | GNN | 0.3 | **<0.25** |
| 8. Bipolar disorder | 14 (6; 8) | 0^i^ | 0.9999-0.9999 | 0.0001-0.0001 | - | - | **-** |
| 9. Insomnia | 1,584 (1,563; 21) | 0^j^ | 0.8001‑1.0000 | 0.7429-0.9991 | - | - | >0.25^j^ |
| 10. Chronic pain | 11,937 (3,213; 8,724) | 3,212 | 0.0505-0.8685 | 0.0422-0.7719 | OFR | 0.2 | **<0.25** |
| 11. Epilepsy | 57 (41; 16) | 0^i^ | 0.9995-1.0000 | 0.0000-0.0006 | - | - | **-** |
| 12. Parkinson’s disease | 113 (41; 72) | 0^j^ | 0.0389-1.0000 | 0.0000-0.6519 | - | - | >0.25^j^ |
| 13. Incontinence | 212 (194; 18) | 0^j^ | 0.3348-1.0000 | 0.0000-0.9842 | - | - | >0.25^j^ |
| 14. Gastrointestinal illness^k^ | 4,480 (349; 4,131) | 347 | 0.0238-0.5693 | 0.0000-0.3939 | OFR | 0.3 | **<0.25** |

Values in bold are indicate well-balanced covariates.
Abbreviations: AM, appropriate medication; COPD, chronic obstructive pulmonary disease; GNN, greedy nearest neighbor; OFR, optimal fixed ratio; PIM, potentially inappropriate medication; PS, propensity score.

^a^ First imputed dataset.
^b^ Range, all observations before matching.
^c^ Applied in the extended common support region.
^d^ Standardized differences were calculated for all covariates used in propensity score matching. Standardized differences <0.25 after propensity score matching indicate well balanced covariates.
^e^ Including heart failure, acute coronary syndromes, chronic therapy after myocardial infarction, stroke, and arterial fibrillation.
^f^ GNN was used because OFR matching was only possible with a caliper >0.6.
^g^ Exact matching on treatment of diabetes at baseline (no diabetes, untreated diabetes, diabetes treated with oral antidiabetics, diabetes treated with insulin) was applied.
^h^ Including dementia-associated behavioural and psychologic syndromes, e.g. hallucination, agitation, aggressiveness, depression, and insomnia. ^i^ Matching was not possible because propensity scores of PIM and AM users did not overlap.
^j^ Matching was not applied because it was not possible to produce well-balanced groups despite different methods and calipers tested.
^k^ Including regular use of non-steroidal anti-inflammatory drugs.
Note: PIM and AM exposure was defined according to “The EURO-FORTA (Fit fOR The Aged) List Version 2”.


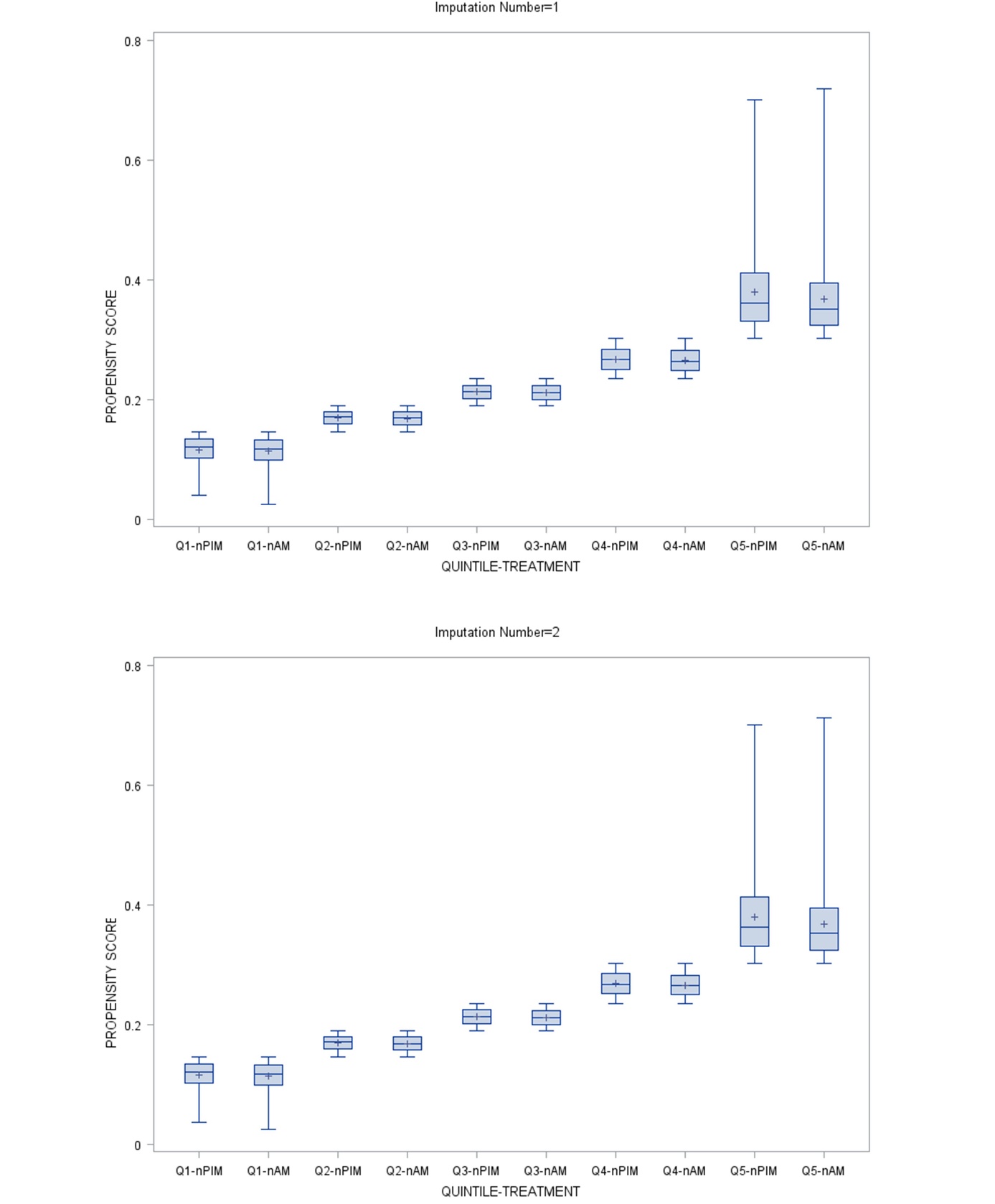


# Figure S1. Distribution of propensity scores for receiving new PIM versus new AM within two years after baseline by quintile and treatment (N=36,534) in 1^st^ and 2^nd^ imputed dataset

Abbreviations: nAM, newly prescribed appropriate medication; nPIM, newly prescribed potentially inappropriate medication; Q, quintile.


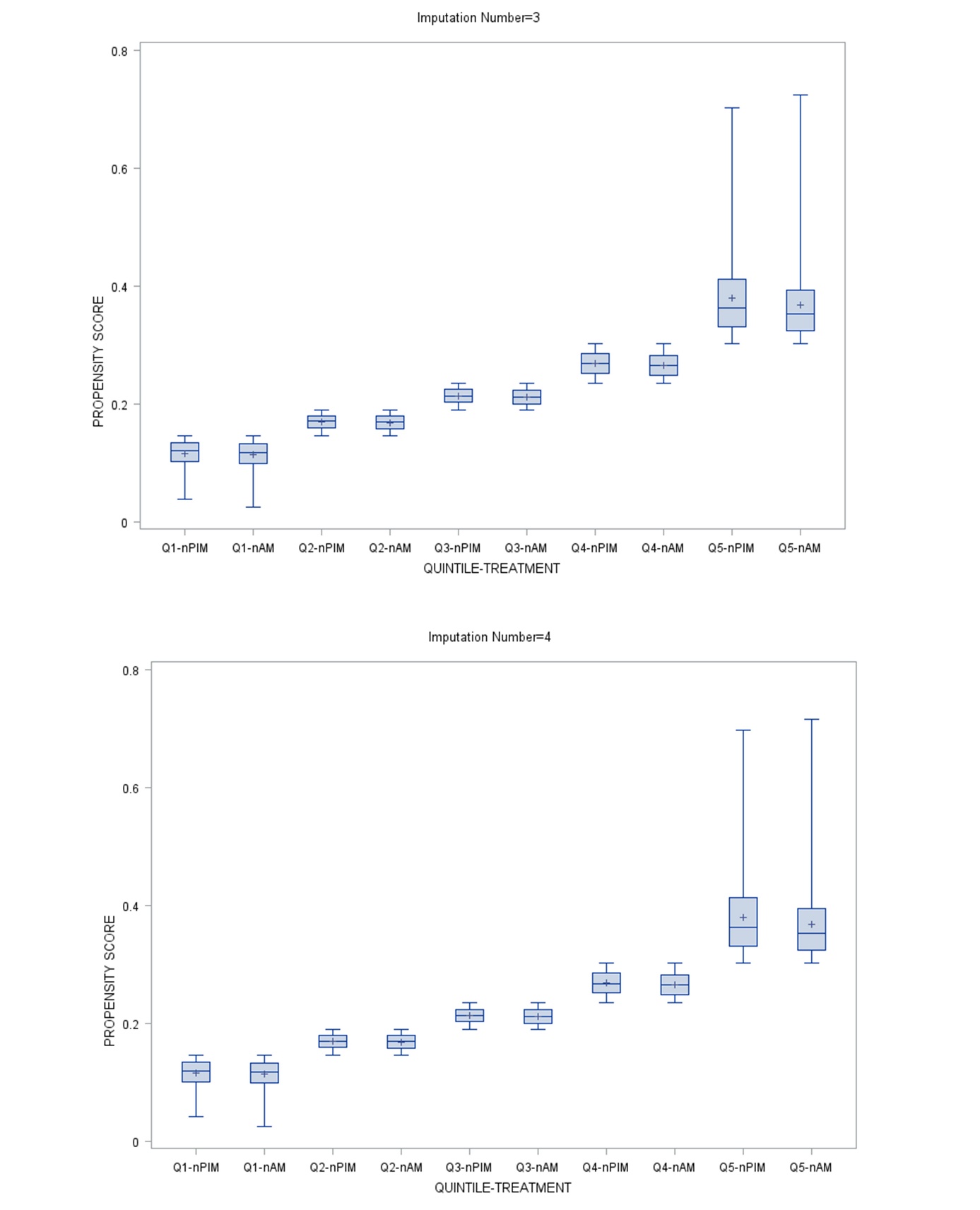


# Figure S2. Distribution of propensity scores for receiving new PIM versus new AM within two years after baseline by quintile and treatment (N=36,534) in 3^rd^ and 4^th^ imputed dataset

Abbreviations: nAM, newly prescribed appropriate medication; nPIM, newly prescribed potentially inappropriate medication; Q, quintile.


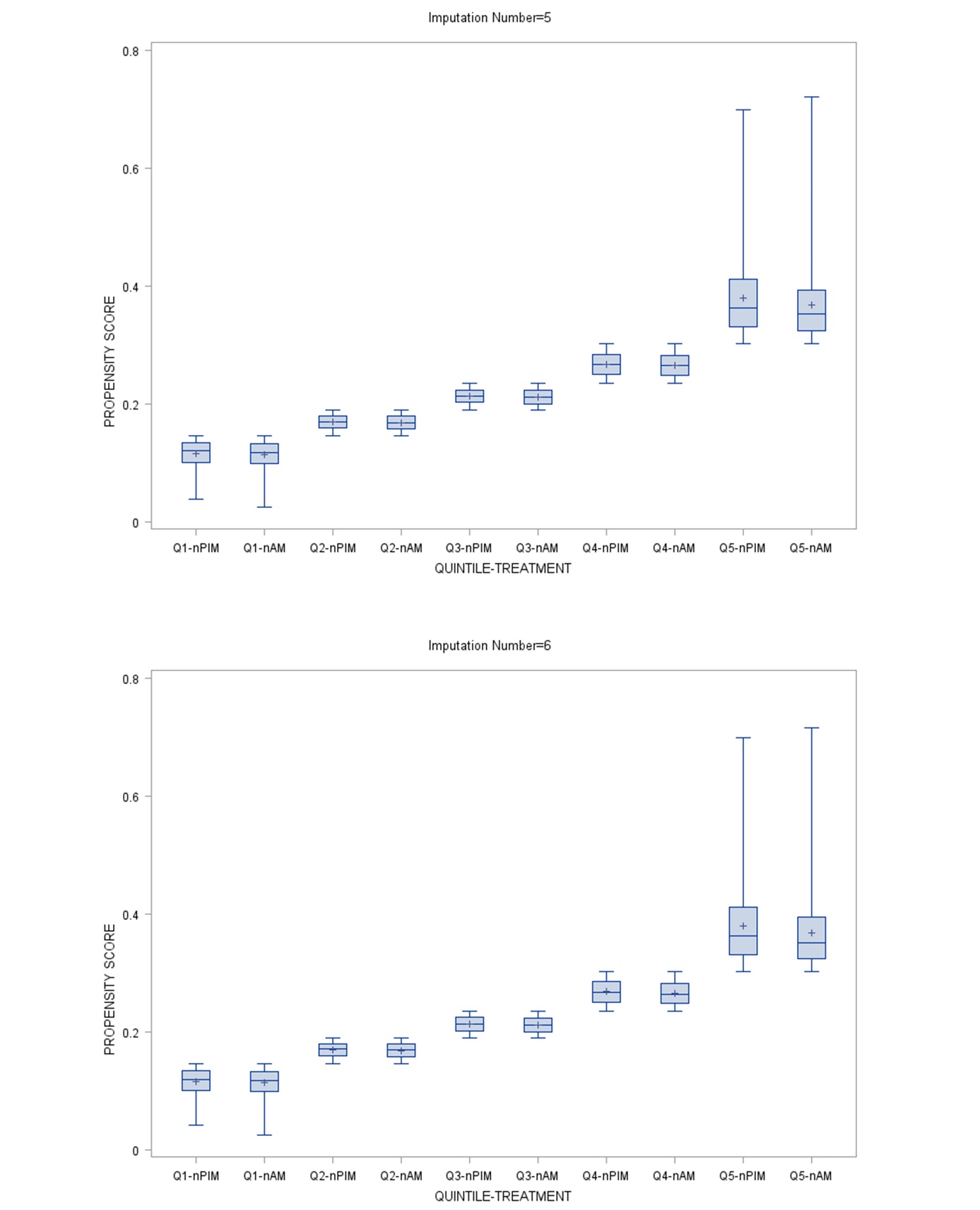


# Figure S3. Distribution of propensity scores for receiving new PIM versus new AM within two years after baseline by quintile and treatment (N=36,534) in 5^th^ and 6^th^ imputed dataset

Abbreviations: nAM, newly prescribed appropriate medication; nPIM, newly prescribed potentially inappropriate medication; Q, quintile.


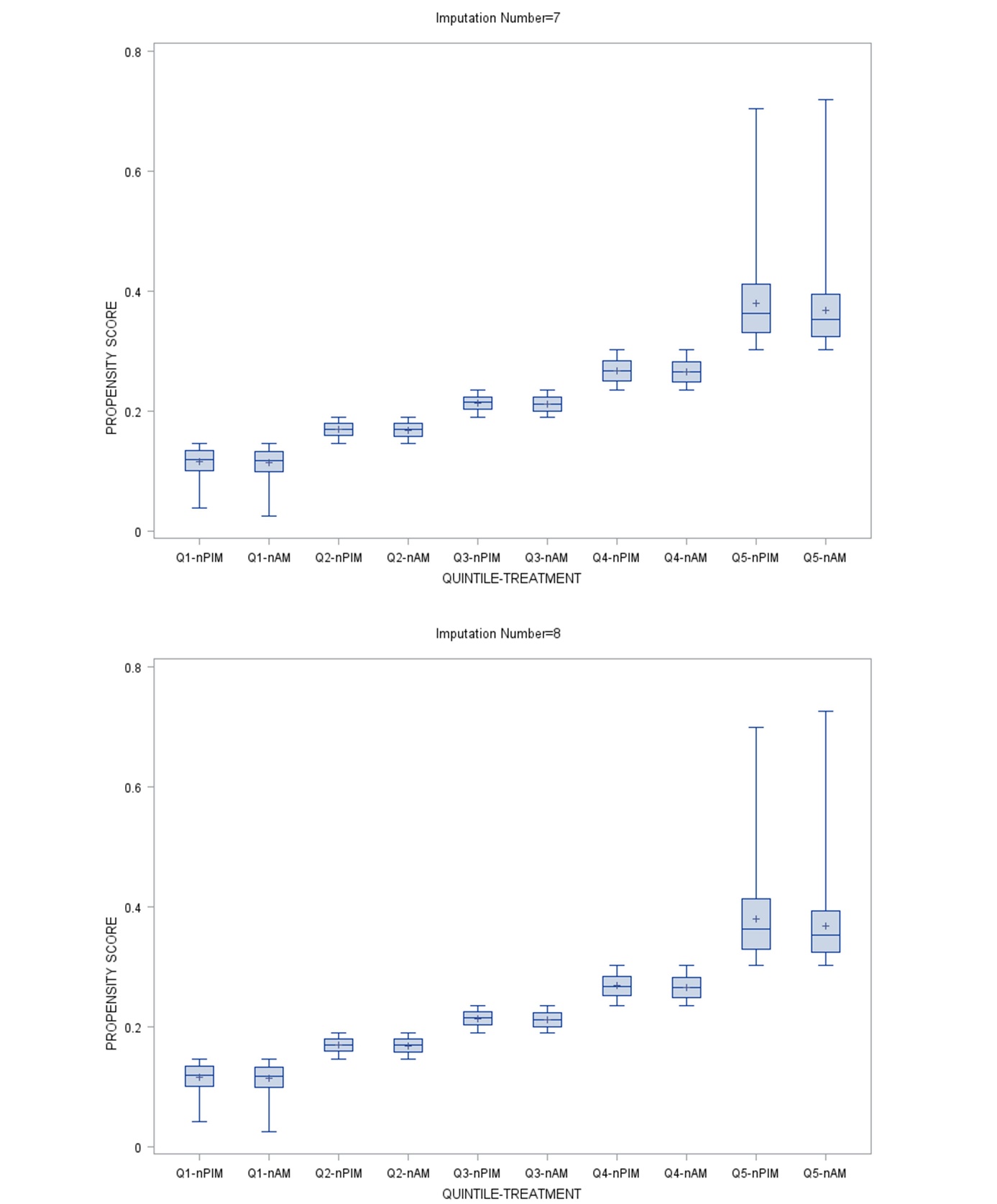


# Figure S4. Distribution of propensity scores for receiving new PIM versus new AM within two years after baseline by quintile and treatment (N=36,534) in 7^th^ and 8^th^ imputed dataset

Abbreviations: nAM, newly prescribed appropriate medication; nPIM, newly prescribed potentially inappropriate medication; Q, quintile.


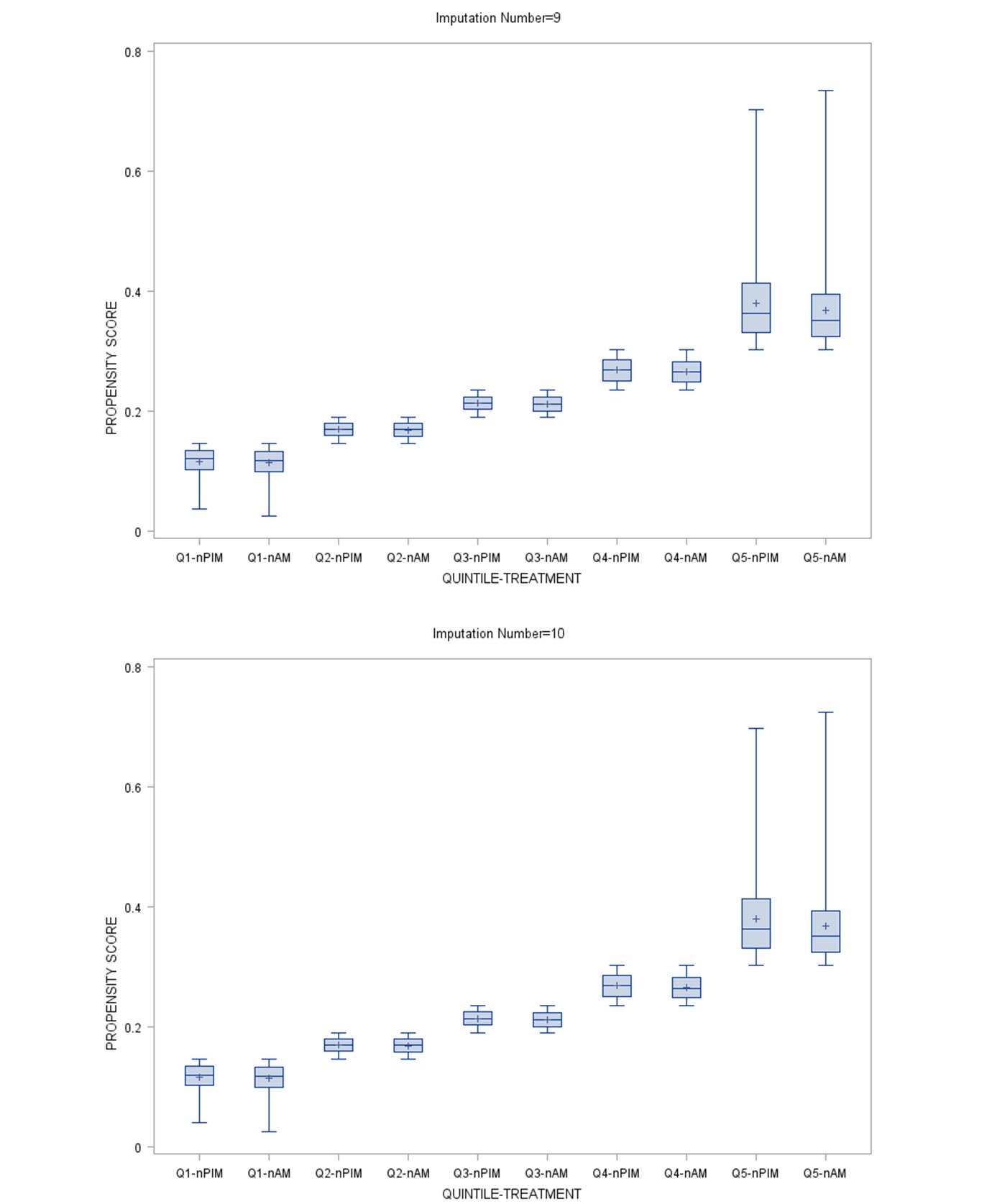


# Figure S5. Distribution of propensity scores for receiving new PIM versus new AM within two years after baseline by quintile and treatment (N=36,534) in 9^th^ and 10^th^ imputed dataset

Abbreviations: nAM, newly prescribed appropriate medication; nPIM, newly prescribed potentially inappropriate medication; Q, quintile.

# Table S4. Assessment of the balance of baseline covariates between new PIM and new AM users after propensity score adjustment (N=36,354)

| **Baseline covariates** | **Standardized difference (mean)^a,b^** |
| --- | --- |
| ***Sociodemographic/ -economic factors*** | **-** |
| Sex |  |
| Female | **0.03** |
| Male |  |
| Age | **0.02** |
| Income^c^ | **-** |
| Very low | **0.02** |
| Low | **0.01** |
| Middle | **0.00** |
| High | **0.01** |
| Very high | **0.01** |
| Years of schooling | **-** |
| ≤10 | **0.02** |
| 11 - <14 | **0.00** |
| ≥14 | **0.02** |
| ***Lifestyle factors*** |  |
| BMI *(kg/m^2^)* | **0.01** |
| Physical activity (MET‑h/week) *median (IQR)* | **<.01** |
| Alcohol consumption, g ethanol/d | **0.04** |
| Smoking status | **-** |
| Never smoker | **0.01** |
| Former smoker, years since quit |  |
| <5 years | **0.01** |
| 5-10 years | **0.02** |
| 11-15 years | **<.00** |
| 16-20 years | **0.01** |
| >20 years | **0.02** |
| Current smoker^d^ |  |
| Regular | **0.01** |
| Occasional | **<.01** |
| ***Comorbidities*** | **-** |
| Type II diabetes mellitus | **-** |
| No | **0.02** |
| Untreated | **0.02** |
| Treated with oral antidiabetics^e^ | **0.06** |
| Treated with insulin | **0.02** |
| Arterial hypertension | **0.01** |
| Stroke | **0.01** |
| Myocardial infarction | **0.04** |
| Heart failure | **0.01** |
| Acute coronary syndromes | **0.03** |
| Atrial fibrillation | **0.03** |
| Asthma | **0.03** |
| COPD | **0.01** |
| Arthritis | **0.03** |
| Osteoporosis | **0.03** |
| History of fractures^f^ | **<.01** |
| Pain^g^ | **0.05** |
| Parkinson’s disease | **<.01** |
| Dementia | **0.02** |
| Depression | **0.04** |
| Bipolar disorder | **0.01** |
| Insomnia, sleep disorder | **0.10** |
| Epilepsy | **0.01** |
| Incontinence | **0.02** |
| Gastrointestinal illness^h^ | **0.04** |
| Anemia | **0.01** |
| History of cancer | **0.01** |
| Number of medications | **0.02** |
| Overall health rating^i^ | **-** |
| Excellent | **0.01** |
| Good | **0.01** |
| Fair | **0.02** |
| Poor | **0.03** |
| ***Functional assessments*** |  |
| Systolic blood pressure *(mmHg)* | **0.04** |
| Diastolic blood pressure *(mmHg)* | **0.03** |
| Grip strength *(kg)* | **0.04** |
| eGFR^j^ *(ml/min/1.73m²)* | **<.01** |
| ***Blood-based biomarkers*** |  |
| CRP *(mg/L)* | **0.02** |
| HDL *(mmol/L)* | **<.01** |
| LDL *(mmol/L)* | **0.01** |
| HbA_1c_ *(mmol/L)* | **0.01** |

Values in bold are indicate well-balanced covariates.
Abbreviations: AM, appropriate medication; BMI, body mass index; COPD, chronic obstructive pulmonary disease; CRP, c-reactive protein; eGFR, estimated glomerular filtration rate; HbA_1c_, haemoglobin A_1c_; HDL, high-density lipoprotein; LDL, low-density lipoprotein, MET, metabolic equivalent of task, PIM, potentially inappropriate medication.

^a^ Calculated from first imputed dataset.
^b^ Standardized differences <0.25 after propensity score adjustment indicate well balanced covariates.
^c^ Average total household income before tax. Categories: very low: <18,000£, low: 18,000-30,999£, middle: 31,000-51,999£, high: 52,000-100,000£, very high: >100,000£.
^d^ Regular smoking was defined as minimum one cigarette, cigar, pipe, etc. per day.
^e^ Including parenteral formulations of glucagon-like-peptide-1 receptor agonists.
^f^ Fractures in last five years.
^g^ Combination of pain-related diagnoses (including migraine, other headache syndromes, facial pain, chronic backpain, chronic pain syndromes, arthritis, osteoarthritis, urinary tract stones, and gout) and self-reported pain in the last month.
^h^ Including regular use of non-steroidal anti-inflammatory drugs.
^i^ Self-reported.
^j^ Calculated based on CKD-EPI equation.

# Table S5. Baseline characteristics of propensity score matched new PIM and AM users

| **Characteristics** | **New AM users  (n=5,906)** | |  | **New PIM users  (n=5,906)** | |
| --- | --- | --- | --- | --- | --- |
|  | **N (%)^a^** | **Median (IQR)^a^** |  | **N (%)^a^** | **Median (IQR)^a^** |
| ***Sociodemographic/ -economic factors*** |  |  |  |  |  |
| Sex | - | - |  | - | - |
| Female | 3,365 (57.0) |  |  | 3,419 (57.9) |  |
| Male | 2,541 (43.0) |  |  | 2,587 (42.1) |  |
| Age^b^ |  | 65 (62; 67) |  |  | 65 (63; 67) |
| Income^c^ | - | - |  | - | - |
| Very low | 2,668 (45.2) |  |  | 2,601 (44.0) |  |
| Low | 1,921 (32.5) |  |  | 1,959 (33.2) |  |
| Middle | 861 (14.6) |  |  | 916 (15.5) |  |
| High | 391 (6.6) |  |  | 363 (6.1) |  |
| Very high | 65 (1.1) |  |  | 67 (1.1) |  |
| Years of schooling | - | - |  | - | - |
| ≤10 | 3,667 (62.1) |  |  | 3,669 (62.1) |  |
| 11 - <14 | 738 (12.5) |  |  | 727 (12.3) |  |
| ≥14 | 1,501 (25.4) |  |  | 1,510 (25.6) |  |
| ***Lifestyle factors*** |  |  |  |  |  |
| BMI *(kg/m^2^)* |  | 27.8 (25.1; 31.1) |  |  | 27.7 (25.0; 30.9) |
| Physical activity *(MET-min/week)* |  | 2,000 (996; 4,066) |  |  | 2,000 (989; 4,260) |
| Alcohol consumption *(g/day)* |  | 11.3 (0.0; 28.3) |  |  | 11.3 (0; 27.6) |
| Smoking status | - | - |  | - | - |
| Never smoker | 2,692 (45.6) |  |  | 2,730 (46.2) |  |
| Former smoker, years since quit |  |  |  |  |  |
| <5 years | 2,274 (38.5) |  |  | 2,248 (38.1) |  |
| 5-10 years | 49 (0.8) |  |  | 46 (0.8) |  |
| 11-15 years | 38 (0.6) |  |  | 38 (0.6) |  |
| 16-20 years | 49 (0.8) |  |  | 43 (0.7) |  |
| >20 years | 270 (4.6) |  |  | 262 (4.4) |  |
| Current smoker^d^ |  |  |  |  |  |
| Regular | 385 (6.5) |  |  | 403 (6.8) |  |
| Occasional | 149 (2.5) |  |  | 136 (2.3) |  |
| ***Comorbidities*** |  |  |  |  |  |
| Diabetes | - | - |  | - | - |
| No | 5,156 (87.3) |  |  | 5,175 (87.6) |  |
| Untreated | 164 (2.8) |  |  | 150 (2.5) |  |
| Treated with oral antidiabetics^e^ | 447 (7.6) |  |  | 441 (7.5) |  |
| Treated with insulin | 139 (2.4) |  |  | 140 (2.4) |  |
| Arterial hypertension | 2,825 (47.8) |  |  | 2,843 (48.1) |  |
| Stroke | 271 (4.6) |  |  | 251 (4.2) |  |
| Myocardial infarction | 679 (11.5) |  |  | 662 (11.2) |  |
| Heart failure | 116 (2.0) |  |  | 108 (1.8) |  |
| Acute coronary syndromes | 1,338 (22.7) |  |  | 1,362 (23.1) |  |
| Atrial fibrillation | 307 (5.2) |  |  | 322 (5.5) |  |
| Asthma | 1,055 (17.9) |  |  | 1,031 (17.5) |  |
| COPD | 963 (16.3) |  |  | 964 (16.3) |  |
| Arthritis | 1,747 (29.6) |  |  | 1,776 (30.1) |  |
| Osteoporosis | 495 (8.4) |  |  | 470 (8.0) |  |
| History of fractures^f^ | 697 (11.8) |  |  | 664 (11.2) |  |
| Pain^g^ | 5,485 (92.4) |  |  | 5,439 (92.1) |  |
| Parkinson’s disease | 23 (0.4) |  |  | 21 (0.4) |  |
| Dementia | 43 (0.7) |  |  | 46 (0.8) |  |
| Depression | 1,146 (19.4) |  |  | 1,100 (18.6) |  |
| Bipolar disorder | 25 (0.4) |  |  | 34 (0.6) |  |
| Insomnia | 2,349 (39.8) |  |  | 2,320 (39.3) |  |
| Epilepsy | 44 (0.7) |  |  | 51 (0.9) |  |
| Gastrointestinal illness^h^ | 2,749 (46.6) |  |  | 2,758 (46.7) |  |
| Incontinence | 159 (2.7) |  |  | 154 (2.6) |  |
| Anaemia | 320 (5.4) |  |  | 294 (5.0) |  |
| History of cancer | 964 (16.3) |  |  | 951 (16.1) |  |
| Overall health rating^i^ | - | - |  | - | - |
| Excellent | 438 (7.4) |  |  | 417 (7.1) |  |
| Good | 2,988 (50.6) |  |  | 3,020 (51.1) |  |
| Fair | 1,943 (32.9) |  |  | 1,944 (32.9) |  |
| Poor | 537 (9.1) |  |  | 525 (9.0) |  |
| ***Functional assessments*** |  |  |  |  |  |
| Systolic blood pressure *(mmHg)* |  | 144 (132; 158) |  |  | 144 (132; 158) |
| Diastolic blood pressure *(mmHg)* |  | 82 (75; 89) |  |  | 82 (75; 89) |
| Grip strength *(kg)* |  | 28.0 (21.0; 37.0) |  |  | 28.0 (21.0; 36.0) |
| eGFR^j^ *(ml/min/1.73m²)* |  | 87.7 (75.9; 94.1) |  |  | 88.1 (76.8; 94.0) |
| ***Blood-based biomarkers*** |  |  |  |  |  |
| CRP *(mg/L)* |  | 1.7 (0.9; 3.5) |  |  | 1.8 (0.9; 3.5) |
| HDL *(mmol/L)* |  | 1.4 (1.1; 1.6) |  |  | 1.4 (1.1; 1.6) |
| LDL *(mmol/L)* |  | 3.4 (2.7; 4.1) |  |  | 3.4 (2.7; 4.1) |
| HbA_1c_ *(mmol/L)* |  | 36.7 (34.2; 39.9) |  |  | 36.8 (34.2; 40.0) |

Abbreviations: AM, appropriate medication; BMI, body mass index; COPD, chronic obstructive pulmonary disease; CRP, c-reactive protein; eGFR, estimated glomerular filtration rate; HbA_1c_, haemoglobin A_1c_; HDL, high-density lipoprotein; IQR, interquartile range; LDL, low-density lipoprotein, MET, metabolic equivalent of task; PIM, potentially inappropriate medication.

^a^ Calculated from first imputed dataset.
^b^ At index prescription.
^c^ Average total household income before tax. Categories: very low: <18,000£, low: 18,000-30,999£, middle: 31,000-51,999£, high: 52,000-100,000£, very high: >100,000£.
^d^ Regular smoking was defined as minimum one cigarette, cigar, pipe, etc. per day.
^e^ Including parenteral formulations of glucagon-like-peptide-1 receptor agonists.
^f^ Fractures in last five years.
^g^ Combination of pain-related diagnoses (including migraine, other headache syndromes, facial pain, chronic backpain, chronic pain syndromes, arthritis, osteoarthritis, urinary tract stones, and gout) and self-reported pain in the last month.
^h^ Including regular use of non-steroidal anti-inflammatory drugs.
^i^ Self-reported.
^j^ Calculated based on CKD-EPI equation.
Note: PIM exposure was defined according to “The EURO-FORTA (Fit fOR The Aged) List Version 2”.

# Table S6. The associations of prevalent PIM use including chronic use of NSAIDs (n=60,403) versus non-use (n=156,708) with hospitalization and all-cause mortality (n=217,111)

| **Outcome** | **N_Event_ (%)^a^** | **Model 1^b^** |  | **Model 2^c^** |  |
| --- | --- | --- | --- | --- | --- |
|  | **PIM users vs. non-users** | **HR (95% CI)** |  | **HR (95% CI)** | **p** |
| **Hospitalization** |  |  |  |  |  |
| 1 month | 193 (0.3) vs. 451 (0.3) | 1.12 (0.94; 1.32) |  | 1.06 (0.86; 1.31) | 0.581 |
| 3 months | 389 (0.6) vs. 937 (0.6) | 1.08 (0.96; 1.22) |  | 0.98 (0.85; 1.14) | 0.882 |
| 6 months | 1,306 (2.2) vs. 3,016 (1.9) | **1.12 (1.05 1.20)** |  | 0.97 (0.89; 1.05) | 0.393 |
| 1 year | 2,805 (4.6) vs. 6,504 (4.2) | **1.12 (1.07; 1.17)** |  | 0.95 (0.90; 1.01) | 0.080 |
| 2 years | 5,857 (9.7) vs. 13,599 (8.7) | **1.12 (1.09; 1.16)** |  | 0.95 (0.91; 0.99) | 0.008 |
| 3 years | 9,069 (15.0) vs. 21,074 (13.5) | **1.12 (1.10 1.15)** |  | 0.96 (0.93; 0.99) | 0.006 |
| 4 years | 12,405 (20.5) vs. 29,243 (18.7) | **1.11 (1.09; 1.13)** |  | **0.96 (0.93; 0.98)** | **0.001** |
| **All‑cause mortality^d^** |  |  |  |  |  |
| 6 months | 110 (0.2) vs. 142 (0.1) | **1.97 (1.53; 2.52)** |  | 1.05 (0.77; 1.43) | 0.779 |
| 1 year | 262 (0.43) vs. 373 (0.2) | **1.77 (1.52; 2.08)** |  | 1.00 (0.82; 1.22) | 0.962 |
| 2 years | 657 (1.1) vs. 993 (0.6) | **1.67 (1.51; 1.84)** |  | 0.92 (0.81; 1.03) | 0.164 |
| 3 years | 1,141 (1.9) vs. 1,778 (1.1) | **1.62 (1.51; 1.75)** |  | 0.93 (0.84; 1.02) | 0.106 |
| 4 years | 1,668 (2.8) vs. 2,682 (1.7) | **1.57 (1.48; 1.67)** |  | 0.94 (0.87; 1.02) | 0.137 |

Values in bold are statistically significant (p<0.05).
Abbreviations: CI, confidence interval; HR, hazard ratio; NSAIDs, non-steroidal anti-inflammatory drugs; PIM, potentially inappropriate medication.

^a^ First imputed dataset.
^b^ Adjusted for age and sex.
^c^ Adjusted for a propensity score, including age, sex, income, years of education, body mass index, physical activity, alcohol consumption, smoking status, number of medications, comorbidities (including arterial hypertension, diabetes, stroke, asthma, COPD, arthritis, heart failure, myocardial infarction, atrial fibrillation, acute coronary syndrome, epilepsy, depression, bipolar disorder, Parkinson’s disease, dementia, chronic pain, osteoporosis, anaemia, history of cancer), fractures in last 5 years, overall health ranking, eGFR, HDL, LDL, HbA1c, CRP, systolic blood pressure, diastolic blood pressure, and grip strength.
^d^ Three- and six-month mortality are not shown because the total number of events was <100.
Note: PIM exposure was defined according to “The EURO-FORTA (Fit fOR The Aged) List Version 2”.
